# Polymeric microwave rectifiers enabled by monolayer-thick ionized donors

**DOI:** 10.1126/sciadv.adv9952

**Published:** 2025-09-19

**Authors:** Nobutaka Osakabe, Jeongeun Her, Takahiro Kaneta, Akiko Tajima, Elena Longhi, Kan Tang, Kazuhiro Fujimori, Stephen Barlow, Seth R. Marder, Shun Watanabe, Jun Takeya, Yu Yamashita

**Affiliations:** ^1^ Material Innovation Research Center (MIRC) and Department of Advanced Materials Science, Graduate School of Frontier Sciences, The University of Tokyo, 5-1-5 Kashiwanoha, Kashiwa, Chiba 277-8561, Japan.; ^2^ Research Center for Materials Nanoarchitectonics (MANA), National Institute for Materials Science (NIMS), 1-1 Namiki, Tsukuba, Ibaraki 205-0044, Japan.; ^3^ School of Chemistry and Biochemistry and Center for Organic Photonics and Electronics, Georgia Institute of Technology, Atlanta, GA 30332-0400, USA.; ^4^ Renewable and Sustainable Energy Institute, University of Colorado Boulder, Boulder, CO 80309, USA.; ^5^ Faculty of Environmental, Life, Natural Science and Technology, Okayama University, 3-1-1 Tsushima-naka, Kita-ku, Okayama 700-8530, Japan.; ^6^ Departments of Chemistry and of Chemical and Biological Engineering, University of Colorado, Boulder, CO 80309, USA.

## Abstract

Solution processing of polymeric semiconductors provides a facile way to fabricate functional diodes. However, energy barriers at metal-semiconductor interfaces often limit their performance. Here, we report rectifying polymer diodes with markedly modified energy-level alignments. The gold electrode surface was treated with a dimeric metal complex, which resulted in a shallow work function of 3.7 eV by forming a monolayer-thick ionized donor layer. When a polymeric semiconductor was coated on the treated electrode, most of the ionized donors remained at the metal-semiconductor interface. The confined ionized donors with the ideal thickness enabled fabrication of a polymer diode with a forward current density of over 100 A cm^−2^. Furthermore, a power conversion efficiency of 7.9% was observed for rectification at a microwave frequency of 920 MHz, which is orders of magnitude higher than that reported for organic diodes. Our findings will pave a way to solution-processed high-frequency and high-power devices.

## Teaser

A doped interface enables high-speed organic rectifiers, achieving efficient operation in the GHz range.

## INTRODUCTION

Solution processing of polymeric semiconductors provides a facile way to fabricate vertical structures for high-power-density devices, including photovoltaic cells (*1*), light-emitting diodes (*2*, *3*), and rectifiers (*4*, *5*). The advantages of polymers for this purpose include tunable electronic properties by molecular design (*6*, *7*) and high uniformity of thin films that suppress leakage problems. One of the target vertical devices based on polymeric semiconductors is a rectifying diode (*5*) that operates AC-to-DC power conversion. Microwave rectifiers serve as power supplies to wireless devices, which is essential for the development of device networks by leveraging the advantages of flexible printed electronics (*8*). Although pioneering works have demonstrated the rectification of voltages above 1 GHz (*9*, *10*), the AC-to-DC power conversion efficiency needs to be considered for wireless powering and communications. This value has not been reported for polymer diodes, and estimated to be low (<0.1%) based on available data on the literature as shown later. This raises questions regarding their practical applications considering the ideal half-wave rectification efficiency (40.5%).

Advanced interface engineering is of key importance to develop efficient polymer diodes for energy harvesting and power management. Depending on the application, either hole or electron transport, or both, need to be considered. For rectifying diodes, transport of only one type of carrier is sufficient for device operation. The active material can be selected to facilitate vertical carrier transport, for which the semicrystalline electron-transporting polymer P(NDIOD-T2) (*11*) is a promising candidate due to its favorable face-on orientation (*12*). On the basis of the typical electron-transporting energy levels of polymeric semiconductors including P(NDIOD-T2), a shallow work function of electrodes (<4 eV) is necessary to decrease the energy barriers at the electrode/polymer interfaces. Because of the instability of shallow-work-function metals, such as Ca and Mg, molecular modifications using self-assembled monolayers (SAMs) and interlayers have been used to tune the work function of electrodes (*13*–*15*). Although polyethyleneimine (PEI) has been reported to achieve a remarkably shallow work function (*16*) among interlayers, there seem to be considerable energy barriers between polymers and electrodes modified with PEI (*4*). This may be partly due to the low carrier concentrations and high trap density of states in polymers (*1*). In addition, the additional resistance of the interlayers can limit the attainable current density considering the exponentially increasing resistance with the thickness of insulating layers on electrodes (*17*).

The use of redox agents and molecular electrical doping has emerged as a powerful tool for modifying energy-level alignments (*3*, *9*, *18*–*24*), and may offer an ideal solution to the above problems. Redox reactions between molecules and metal electrodes result in the formation of charged layers and shifts in work function (*25*, *26*). While most redox agents do not have sufficient reducing strength and stability to achieve a low work function below 4 eV, dimeric donor molecules have been demonstrated to realize such low work functions (*27*, *28*). This work function shift is predicted to occur with a monolayer-thick ionized donor layer on electrodes (*29*), suggesting the possibility of forming ideally thin and effective interlayers. However, it has been unclear whether such a method is applicable to fabricate polymer diodes, where the vertical stacking structures need to be controlled during the solution process. The diffusion of donor and acceptor molecules has been a critical problem in the fabrication of advanced polymer devices (*18*, *30*).

**Fig. 1.**
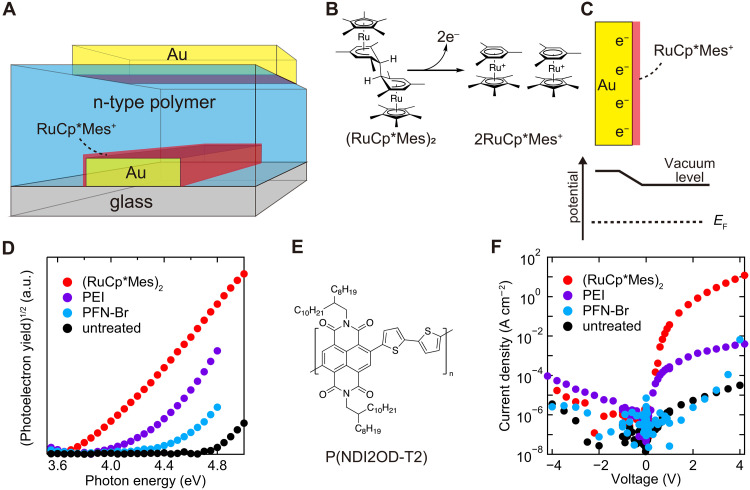
Polymer diodes using surface treatments of bottom electrodes. (**A**) A schematic illustration of a polymer diode using (RuCp*Mes)_2_ treatment on the bottom electrode. (**B**) Illustrations of redox reactions of (RuCp*Mes)_2_ and (**C**) a model describing the shift in work function through (RuCp*Mes)_2_ surface treatment. (**D**) PYS measurements of Au electrodes treated with (RuCp*Mes)_2_ and conventional interlayers. The names of materials used are denoted as the legends. (**E**) The molecular structure of P(NDIOD-T2). (**F**) The current density-voltage characteristics of the fabricated polymer diodes with various treatments on the bottom electrodes. The diodes had an area of 2500 μm^2^ and a typical capacitance of 0.9 pF.

In this study, we demonstrate polymeric microwave rectifiers using the treatment of metal surfaces with dimeric donor molecules (Fig. 1, A and B). In our method, gold electrodes were exposed to a solution of the dimeric organometallic complex donor molecule (*31*), which decreased the work function of the gold electrode to 3.7 eV even after air exposure. X-ray photoelectron measurements suggest that the cationic donor molecules were present on the treated gold surface with a thickness close to one monolayer. To fabricate vertical diodes, solution-processed n-type polymeric semiconductor thin films were fabricated on the treated gold electrodes. Owing to the cationic donor layer present only around the bottom electrode, high forward current density and rectifying ratios were achieved, leading to power conversion efficiency of 7.9% at 920 MHz. Notably, DC output power of 5 mW was obtained, which is orders of magnitude higher than previous reports. Our findings will pave a way to high-speed and high-power vertical polymer devices.

## RESULTS

### Diodes using surface-treated electrodes

To achieve the surface treatment of gold electrodes, the solution of donor molecule was spin-coated onto the electrodes followed by washing with pure solvents. The donor molecule (RuCp*Mes)_2_ (Cp* is pentamethylcyclopentadienyl and Mes is 1,3,5-trimethylbenzene) was chosen owing to its high reducing capability through cleavage and electron transfer reactions to form RuCp*Mes^+^ (*31*). Figure 1 (B and C) shows a possible model of the (RuCp*Mes)_2_ treatment, where (RuCp*Mes)_2_ and gold surface undergo redox reactions. This leaves cationic donor layers on the negatively charged gold surface, forming a dipole that would decrease the work function similar to what is reported in previous literature (*27*–*29*).

The work functions of the electrodes were evaluated by photoelectron yield spectroscopy (PYS). Compared to the untreated gold electrode, the electrode treated with (RuCp*Mes)_2_ showed smaller threshold photon energies to give rise to photoelectron yields, which confirms a decrease in the work function (Fig. 1D). We also compared the treatments with commonly used interlayers of PEI and poly[9,9-bis(3′-(*N*,*N*-dimethyl)-*N*-ethylammoinium-propyl-2,7-fluorene)-alt-2,7-(9,9-dioctylfluorene)]dibromide (PFN-Br). In our measurements, the (RuCp*Mes)_2_ treatment resulted in a work function of 3.7 eV, which is lower than the values obtained with either PEI or PFN-Br. This value is sufficiently small compared with the energy level of the lowest unoccupied molecular orbital (LUMO) of the target n-type polymer P(NDIOD-T2) (3.84 eV, Fig. 1E) (*11*, *32*). Please see figs. S1 to S3 for fittings of PYS measurements. Treatments with other donor molecules, 4-[1,3-dimethyl-2,3-dihydro-1*H*-benzoimidazol-2-yl)phenyl]dimethylamine (N-DMBI-H) and cobaltocene (CoCp_2_), resulted in work function higher than the case with (RuCp*Mes)_2_, which suggests the importance of the high reducing capability of (RuCp*Mes)_2_ (figs. S1 and S2). Note that our measurements were conducted after a short exposure of the samples to air. In addition, a work function of 3.7 eV after the (RuCp*Mes)_2_ treatment was observed even in PYS measurements in air (figs. S1 and S3), which shows moderate ambient stability of the treatments. Considering the low work function of this sample, the observed ambient stability is remarkable (*3*) and may be attributed to the stable (*33*), hydrophobic, and bulky character of the RuCp*Mes^+^.

The injection properties of the surface-treated electrodes were evaluated using polymer diodes. After the treatment of bottom electrodes using the interlayers or (RuCp*Mes)_2_, P(NDIOD-T2) was spin-coated with a thickness of ca. 80 nm (fig. S4), followed by evaporation of the top gold electrodes. Devices with the treated bottom electrodes showed higher current densities under a positive bias, where electrons were injected from the bottom electrodes (Fig. 1F). Furthermore, the maximum current density was higher for (RuCp*Mes)_2_-treated device than for devices using PEI or PFN-Br. Here, a smaller work function should be more advantageous for approaching ohmic contact, considering the LUMO energy level of P(NDIOD-T2) and the trap density of states in polymer thin films (*1*, *34*). This highlights the importance of the robust control of work function by (RuCp*Mes)_2_, which realized larger shifts in work function compared to conventional interlayers or SAM treatments (*13*, *35*). We observed the highest current density of over 10 A cm^−2^ and a rectifying ratio of 10^6^ when the (RuCp*Mes)_2_ treatment was used. These results support the successful confinement of the donor molecule in the vertical direction of solution-processed polymer diodes.

### Surface analysis of the treated gold electrodes

**Fig. 2.**
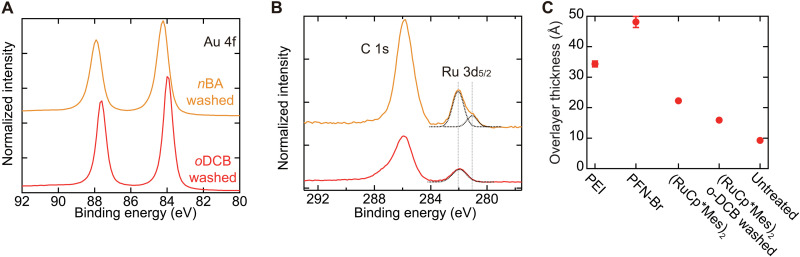
Evaluation of dopant distribution based on XPS measurements. XPS spectra of Au electrodes treated with (RuCp*Mes)_2_ after washing the surface with *n*BA (orange) and further with *o*DCB (red) in the (**A**) Au 4f, (**B**) C 1s, and Ru 3d regions. The black dashed lines show Gaussian peak shapes based on fittings. The gray dashed line shows the position of the peaks for the sample washed with *n*BA. The intensity of spectra was normalized on the basis of the total intensity of Au 4f peaks. The binding energy was shifted so that both spectra have the same binding energy for the C 1s main peaks to clarify the chemical shifts without effects of difference in work functions. (**C**) The overlayer thicknesses on the Au electrodes, which are evaluated on the basis of the Au contributions to XPS spectra. The error bar shows one SD. See text S2 for details of calculation.

The surface elemental composition of the (RuCp*Mes)_2_-treated gold electrode was analyzed using x-ray photoelectron spectroscopy (XPS). The treatment was conducted in the same manner as in the above experiments, where an *n*-butyl acetate (*n*BA) solution of (RuCp*Mes)_2_ was spin-coated on the electrode, followed by washing with pure *n*BA. To understand the effect of the processing solvents, another sample was prepared, which was further washed with *o*-dichlorobenzene (*o*DCB). For the sample after *n*BA washing, Ru 3d_5/2_–derived peaks were observed at 281.1 and 282.0 eV in addition to Au 4f and C 1s peaks (Fig. 2, A and B). The two different Ru 3d_5/2_ peaks originate from complexes with different charges, where the peak at the higher binding energy is ascribed to RuCp*Mes^+^ and the peak at the lower binding energy to (RuCp*Mes)_2_ (*36*). When the electrode surface was further washed with *o*DCB, only the peak originating from RuCp*Mes^+^ was observed. The above results suggest that while neutral (RuCp*Mes)_2_ was washed away by *o*DCB, RuCp*Mes^+^ was bound to the gold surface sufficiently tightly to endure such a solution process.

Fitting of XPS spectra was conducted (see text S1), and the ratios of Ru atomic compositions to the total ones were evaluated to be 2.1% after washing with *n*BA and 0.9% after washing with *o*DCB. The value after washing with *o*DCB is consistent with one assuming a model with a monolayer of RuCp*Mes^+^ covering the gold surface (table. S10). For comparison, the XPS spectra of the gold electrodes with other interlayers and donor molecules were also measured (text S1). On the basis of the atomic composition and an overlayer model (*37*) (text S2), the thickness of the layers on the gold electrode was remarkably smaller for (RuCp*Mes)_2_ treatments (1.6 nm) than for conventional interlayers (over 3 nm, Fig. 2C). This is important considering the exponentially increasing resistance with the thickness of insulating layers on electrodes (*17*). Thus, the (RuCp*Mes)_2_ treatment realizes a marked decrease in the work function while minimizing the thickness and additional resistance of the interlayer.

### Distribution of ionized donors

**Fig. 3.**
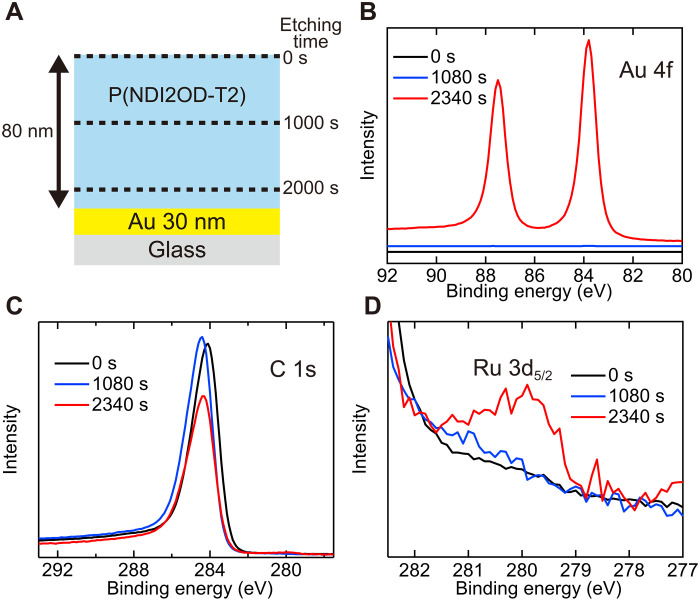
Evaluation of dopant diffusion based on depth profile. (**A**) Illustration of the depth profile measurements using Ar ion gun etching. (**B** to **D**) XPS spectra in Au 4f, C 1s, and Ru 3d_5/2_ regions with varying etching times. The etching times used are denoted as the legends.

The distribution of RuCp*Mes^+^ in the vertical direction was investigated using XPS depth profiles. The *o*DCB solution of P(NDIOD-T2) was spin-coated onto gold electrodes treated with (RuCp*Mes)_2_. As shown in Fig. 3A, an Ar ion gun was used to etch P(NDIOD-T2) and evaluate the atomic composition at varying depths. Figure 3 (B to D) shows representative XPS spectra at etching times of 0, 1080, and 2340 s. The Au 4f peaks in Fig. 3B were observed for an etching time of 2340 s, indicating that most of the P(NDIOD-T2) thin film was etched under this condition, where the gold electrode was close to the top surface. The Ru 3d peak at approximately 280 eV also appeared under this etching condition (Fig. 3D). The above results demonstrate that RuCp*Mes^+^ is present mostly at the interface between P(NDIOD-T2) and the bottom gold electrode in our diode without diffusing into the entire P(NDIOD-T2) thin film.

**Fig. 4.**
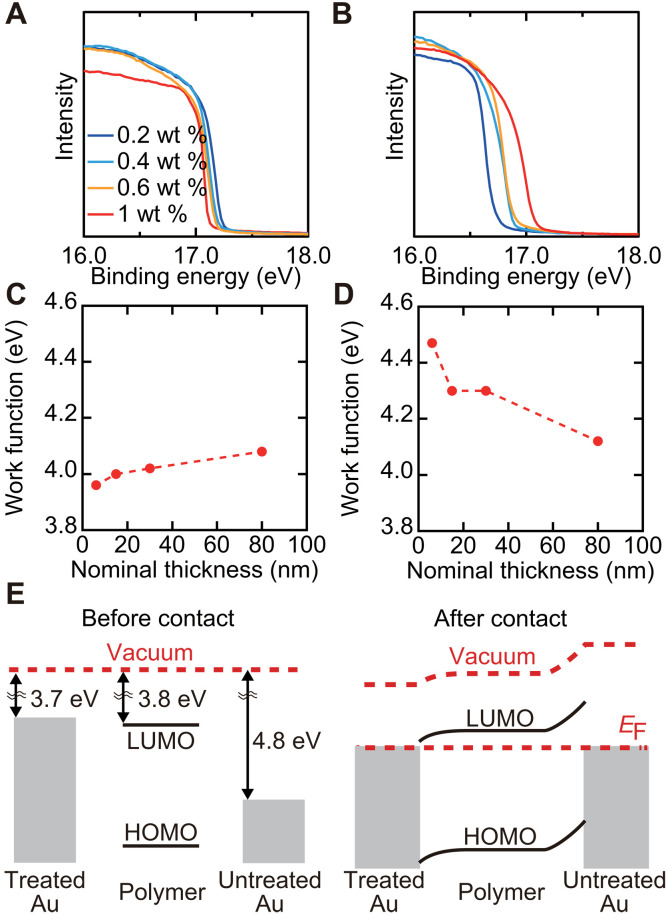
Energy level alignments at metal-semiconductor interfaces. UPS spectra of samples with varying thickness of P(NDIOD-T2) on (**A**) the (RuCp*Mes)_2_-treated and (**B**) untreated gold electrodes around the cut off regions. The concentrations of P(NDIOD-T2) solutions used were denoted as legends. The origins of *x* axis are the Fermi energy. Work functions evaluated for the samples with (**C**) the (RuCp*Mes)_2_-treated and (**D**) untreated gold electrodes. (**E**) Illustrations of the models of energy diagram and band bending before and after the contact. LUMO level of P(NDIOD-T2) polymer is according to the literature (*32*).

In diodes with low injection barriers at the bottom electrode and high injection barriers at the top electrode interfaces, band bending in opposite directions are predicted around the interfaces. This was verified in our diodes by ultraviolet photoelectron spectroscopy (UPS) measurements. Here, we used the (RuCp*Mes)_2_-treated and untreated gold electrodes covered with P(NDIOD-T2) thin films with varying thicknesses. Note that the donor molecules were confined around the electrode interface in the treated samples, which is verified by our XPS measurements of these samples (see text S3). Considering the high surface sensitivity of UPS measurements, UPS measurements of these samples indicate the work function and band bending at different vertical positions (*38*). Figure 4 (A and B) shows the cutoff regions of the UPS spectra. Figure 4 (C and D) shows the work functions evaluated on the basis of the spectra (see text S4 for the details of the analysis). The whole UPS spectra are shown in fig. S20. For the samples with (RuCp*Mes)_2_ treatment, the work function increased with the increasing thickness of P(NDIOD-T2), while the opposite trend was observed for the samples without the treatment. These observations are consistent with the band bending model predicted for a semiconductor layer in contact with low- and high-work-function electrodes (Fig. 4E). The observed band bending extends over tens of nanometers, which is consistent with the previous studies for polymer thin films with low carrier concentrations and trap density of states (*39*, *40*).

### Rectification performance of diodes

**Fig. 5.**
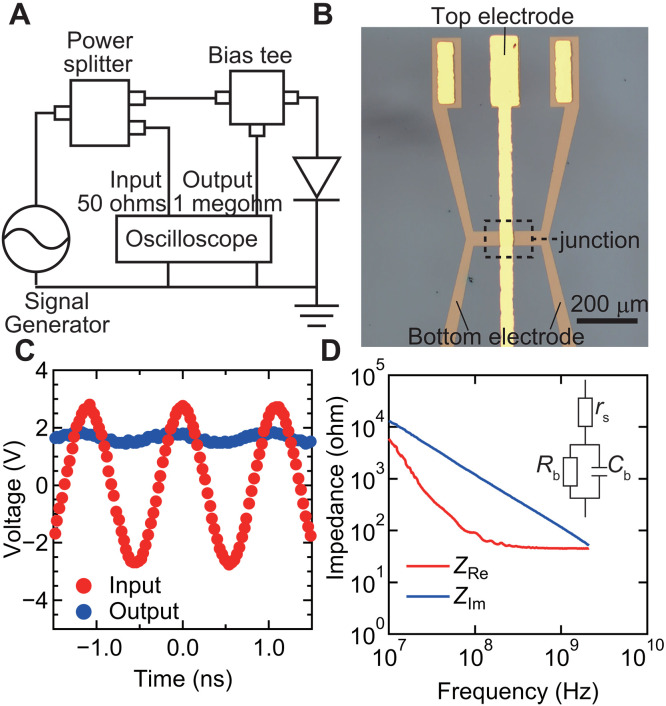
Rectification at the UHF band. (**A**) An illustration of the measurement setup. (**B**) A photo of the fabricated diode using a pattern suitable for measurements at the UHF band. (**C**) The input and output voltages monitored by the oscilloscope using our polymer diode with the (RuCp*Mes)_2_-treated bottom electrode. The input voltage was an AC 920 MHz sine wave. The area of diode was 2500 μm^2^. (**D**) Real and imaginary parts of impedance of our polymer diode with the (RuCp*Mes)_2_-treated bottom electrode. The circuit model of a Schottky diode is shown as the inset. The capacitance of diode was estimated to be 0.85 pF.

The rectification performance of our diode using (RuCp*Mes)_2_ treatment was evaluated at the ultrahigh frequency (UHF) band. In our setup (Fig. 5A), the input power from the signal generator go through a power splitter that distributes ca. 40% of the input power to an oscilloscope and the diode. The output voltage from the diode was monitored by the oscilloscope with 1-megohm termination. Figure 5B shows a photo of the fabricated diode, which is designed to be measured with a high-frequency ground-signal-ground (GSG) probe. Figure 5C shows the results obtained when the input signal was 920 MHz and 16 dBm. Under this condition, the input AC voltage monitored by the oscilloscope was ca. 6 V peak-to-peak ( $V_{\text{pp}}$ ) and the output DC voltage from the diode was ca. 1.6 V. This is in contrast to previous reports on polymer diodes, whose DC output voltages were less than 0.2 V at 1 GHz (*9*), and demonstrates that our diode converts UHF AC voltages to DC voltages with a small voltage loss under the above condition.

To operate diodes at frequencies above the HF band, we considered requirements on device parameters. The first requirement is a sufficiently short RC delay, where the diode needs to possess low capacitance and resistance. This will be examined in the following section. The second requirement is that the semiconductor has sufficiently high mobility and low thickness that charge carriers can travel through the thin film (*41*). Vertical structures are advantageous for fulfilling this requirement, where the required mobility is the order of 0.1 cm^2^ V^−1^ s^−1^ when the thickness is 100 nm scale (See text S5 for details). This value may be attainable with semicrystalline P(NDIOD-T2) with preferential face-on-oriented packing (*12*).

To understand the RC delay and circuit model of our diode at high frequencies, the one-port scattering parameters were measured to evaluate real ( $Z_{\text{Re}}$ ) and imaginary ( $Z_{\text{Im}}$ ) parts of impedance (Fig. 5D). Considering the RC delay, the power efficiency of the circuit will start to markedly decrease when the frequency exceeds a cutoff frequency ( $f_{c}$ ). $f_{c}$ is the frequency of the intersection of $Z_{\text{Re}}$ and $Z_{\text{Im}}$ , which was 2 GHz in our case. The equivalent circuit model of a Schottky diode can be represented by the circuit shown in the inset of Fig. 5D. $r_{s}$ is the sum of the series resistances including the resistances of electrodes. $R_{b}$ is the parallel resistance and $C_{b}$ is the parallel capacitance of the depletion layer. In this case, $C_{b}$ contributes to RC delay (*42*) and was estimated to be rather small value of 0.85 pF from the observed $Z_{\text{Im}}$ . On the basis of the geometry of our device and expected permittivity of the polymer, the depletion layer is 80 nm thick, which is almost identical to the total thickness of the polymer. This is consistent with the XPS measurements showing that dopants are present only around the bottom electrode interface. $r_{s}$ contributes to the RC delay, whose value can be estimated to be equal to $Z_{\text{Re}}$ at high frequencies (*42*). $r_{s}$ was ca. 45 ohms in this device, where the evaluated small capacitance and resistance ensure the short RC delay of our device. Note that, while decrease in $r_{s}$ increases $f_{c}$ , the use of $r_{s}$ close to 50 ohms is desirable considering impedance matching.

**Fig. 6.**
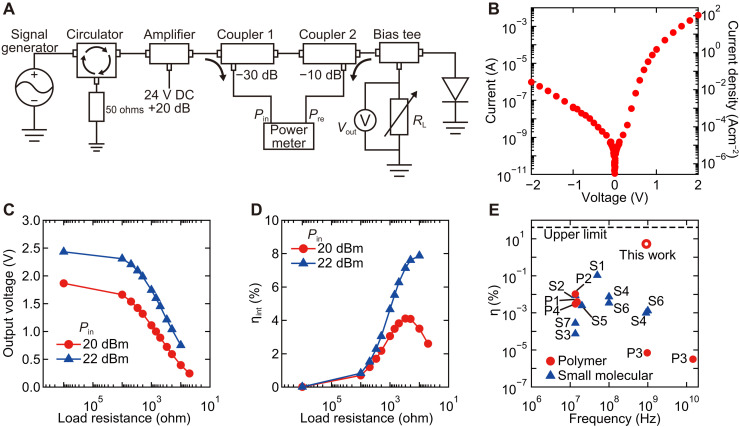
Measurements of AC-to-DC power conversion efficiency. (**A**) An illustration of the measurement setup. (**B**) The DC performance of the used polymer diode with the (RuCp*Mes)_2_-treated bottom electrode and P(NDIOD-T2) layer fabricated using a 0.5 wt % solution. The area of diode was 3750 μm^2^. The capacitance of the diode was 3.3 pF based on the measurement shown in fig. S22. (**C**) The output voltage and (**D**) AC-to-DC power conversion efficiency measured with our diode fabricated using 0.5 wt % P(NDIOD-T2) solution. The AC power from signal generator ( $P_{\text{in}}$ ) were 20 and 22 dBm. (**E**) Comparison of the efficiency to those in literature studies of diodes based on small molecules [S1 (*41*), S2 (*46*), S3 (*47*), S4 (*48*), S5 (*49*), S6 (*10*), and S7 (*50*)] and polymers [P1 (*4*), P2 (*5*), and P3 (*9*)].

We examined whether high output DC power and high AC-to-DC power conversion efficiency ( η ) are possible with our diodes. In this case, a reasonably large DC output voltage ( $V_{\text{out}}$ ) needs to be developed even when the load resistance ( $R_{L}$ ) becomes rather small. η is defined as $P_{\text{out}}/P_{\text{in}}$ , where $P_{\text{in}}$ is the AC input power and $P_{\text{out}}$ is the DC output power. $P_{\text{out}}$ was calculated using $R_{L}$ and $V_{\text{out}}$ . To eliminate the effects of the power reflection that occurs mainly due to impedance mismatch, we also evaluated $\eta_{\text{int}}$ , which is defined as $P_{\text{out}}/P_{\text{in},\text{eff}}$ . The effective input power ( $P_{\text{in},\text{eff}}$ ) and the reflected power ( $P_{\text{re}}$ ) were measured using the setup shown in Fig. 6A, where $P_{\text{in},\text{eff}}=P_{\text{in}}-P_{\text{re}}$ . In these measurements, we used a 0.5 weight % (wt %) P(NDIOD-T2) solution to fabricate a diode, which resulted in a depletion layer thickness of ca. 35 nm (text S6). The capacitance of the diode was 3.3 pF based on the impedance measurement shown in fig. S22. When a 50-ohm system is used, $f_{c}$ of the system becomes 970 MHz, still higher than the used UHF frequency. Figure 6B shows the current-voltage characteristics obtained with this diode, where a current level of over 1 mA and current density of over 100 A cm^−2^ at 2 V forward bias were observed. Even for the thin thickness, our diodes show moderate reproducibility and bias stability as discussed in texts S7 and S8. Figure 6 (C and D) shows $V_{\text{out}}$ and $\eta_{\text{int}}$ at different load resistances. While conventional organic small molecular and polymer diodes showed a DC output of less than 20 μW (*9*, *10*), our diode showed a DC output power reaching 5 mW. In contrast to previous reports, η of our diode reached 5.2%, approaching the ideal efficiency of 40.5% for a half-wave rectifier (Fig. 6E). For details on the efficiency calculation, see text S9. The high rectification performance of our device is ascribed to the high current level at a small bias in the diode. When the DC power is consumed at the load resistor, the DC current needs to pass through the diode and load resistor, where the diode needs to flow a high current with a small voltage loss. From this point of view, the use of diodes with a thin depletion layer thickness is advantageous and our diode outperforms previously reported organic semiconductor diodes based on small molecules and polymers (texts S10 and S11).

## DISCUSSION

Considering all requirements, a microwave rectifying diode needs to be designed so that the junction shows a reasonable rectification ratio, small capacitance, and a high current at a small bias. Impedance matching may be achieved by designing the electrodes and circuits rather than junction of the diode (*43*). To design a diode with a specific combination of materials, the depletion layer thickness and the junction area play key roles. In most organic diodes, while the forward current level is only proportional to the junction area, it shows larger dependence on the depletion layer thickness. For instance, in the space-charge limited current model following the Mott-Gurney’s law, the current is inversely proportional to the cube of the thickness (*44*). Considering this, it is reasonable to decrease the depletion layer thickness to increase the current density and then tune the junction area to fulfill the requirement on the capacitance. Our diode showed the reasonable rectification ratio with the thin depletion layer thickness of ca. 35 nm. Use of efficient donor molecules in such a thin vertical device was the key to achieving the observed high device performance, which highlights advantages of the used processes and materials. Here, the stable and highly reducing donor enabled facile fabrication of the monolayer-thick ionized donor layer, which features marked energy-level alignments, suppressed diffusion of donors, and minimized thickness and additional resistance of the interlayer. The attainable performance may be further enhanced by exploring molecular ions through the ion exchange doping and related technologies to control dopant ions (*20*–*24*).

In this study, the use of dimeric donor molecules in vertical polymer diodes was demonstrated, where a monolayer-thick RuCp*Mes^+^ layer was deposited to the surface of gold electrode. This method decreased the work function of gold electrode to 3.7 eV even after air exposure and effectively modified the energy-level alignment and the band bending when the electrode was in contact with P(NDIOD-T2). The fabricated diode exhibited a high current density of over 100 A cm^−2^ and rectification at 920 MHz. The output DC power reached 5 mW owing to the high current levels at small forward voltages in our device, demonstrating a benchmark efficiency among diodes composed of printable semiconductors. These remarkable device performances owe to the monolayer-thick ionized donors, which feature marked energy-level alignments, suppressed diffusion of donors, and minimized thickness and additional resistance of the interlayer. Our findings will contribute not only to printed microwave rectifiers but also to polymer vertical optoelectronic devices in general, where the control of semiconductor-metal interfaces plays a key role.

## MATERIALS AND METHODS

### Materials

The polymeric semiconductor P(NDIOD-T2) was purchased from Ossila. Batch M1201A3 was used with the following molecular weights: weight-average molecular weight (*M*_w_) = 202,261, number-average molecular weight (*M*_n_) = 90,982, and polydispersity index = 2.22. (RuCp*Mes)_2_ was synthesized as described in the literature (*45*). CoCp_2_ was purchased from Sigma-Aldrich. N-DMBI-H was purchased from Tokyo Chemical Industry Co. Ltd. Anhydrous *o*DCB and *n*BA were purchased from FUJIFILM Wako Pure Chemical Corporation (FUJIFILM Wako). Methanol in the super dehydrated grade was purchased from FUJIFILM Wako. Polyethylenimine was purchased from Sigma-Aldrich in the branched form whose $M_{w}$ is claimed to be 25,000 by light scattering and $M_{n}$ to be 10,000 by gel permeation chromatography (GPC). PFN-Br was purchased Sigma-Aldrich whose $M_{w}$ is claimed to be 30,000 to 50,000 by GPC. EAGLE XG (Corning) glass substrates were used in this study.

### Fabrication of bottom electrodes

Bottom electrodes were fabricated on glass substrates. Three-nanometer Cr and 30-nm Au were thermally deposited. The samples used for the photoelectron measurements used electrodes deposited over the whole surface. The electrodes of the diodes used in Fig. 1 were patterned through shadow masks, and the electrodes of the diodes used in Figs. 5 and 6 were patterned through a lift-off process using a positive photoresist TLOR-P003 (Tokyo Ohka Kogyo Co. Ltd.). After the deposition of bottom electrodes, the samples were cleaned by ultraviolet/O_3_ for 15 min. The samples for diodes were further cleaned by sonication in acetone and isopropanol for 10 min each.

(RuCp*Mes)_2_, CoCp_2_, and N-DMBI-H treatments were conducted in a N_2_ purged glove box. (RuCp*Mes)_2_ and N-DMBI-H were dissolved in *n*BA at a concentration of 2 mM. CoCp_2_ was dissolved in acetonitrile at a concentration of 1 mM. These solutions were dropped on samples. After waiting for 30 s, spin coating at 2000 rpm was conducted. The samples were heated at 80°C for 5 min. Then, the samples were washed by spinning off *n*BA at 2000 rpm, followed by heating at 80°C for 5 min. PEI treatment was conducted in air. Aqueous solution with 0.2 wt % PEI was prepared. This solution was dropped on samples. After waiting for 30 s, spin coating at 2000 rpm was conducted. The samples were heated at 80°C for 5 min. Then, the samples were washed by spinning off pure water at 2000 rpm, followed by heating at 80°C for 5 min. PFN-Br treatment was conducted in a N_2_ purged glove box. Methanol in the super dehydrated grade was used to dissolve PFN-Br (0.5 mg/ml). This solution was dropped on samples. After waiting for 30 s, spin coating at 2000 rpm was conducted. The samples were heated at 80°C for 5 min.

### Fabrication of diodes

Fabrications of P(NDIOD-T2) thin films were conducted in a N_2_ purged glove box. P(NDIOD-T2) was dissolved in *o*DCB. A concentration of 1 wt % was used unless indicated. Experiments shown in Fig. 4 used varying concentrations and ones shown in Fig. 6 used 0.5 wt %. Spin coating was conducted at 2000 rpm for 1 min using the P(NDIOD-T2) solutions preheated at 120°C. Then, samples were dried at 80°C for 20 min. The film thickness of P(NDIOD-T2) coated at a concentration of 1 wt % was determined to be 80 nm using a Dektak Stylus Profiler (Bruker). The surface profile is shown in fig. S4.

Top electrodes for diodes were fabricated by thermal deposition through shadow masks. Thirty-nanometer Au was deposited on the diodes used for Figs. 1 and 5. Thirty-nanometer Au and 300-nm Cu were deposited on ones used for Fig. 6. CYTOP CTL-809M diluted to one-fifth of the original concentration was spin coated on diode devices as an encapsulation layer. The spin coating condition was 2000 rpm for 1 min. Samples were then vacuum dried at room temperature for 5 min.

### XPS and UPS analysis

XPS and UPS measurements were conducted using a KRATOS ULTRA 2 instrument with monochromatic Al Kα x-rays and He I α (21.2 eV). PYS measurements were conducted using SUMITOMO PYS-202 instrument with a xenon lamp. DC electrical measurements were conducted using Keithley 2450.

### Measurement setups for UHF rectification

Followings are the list of components used in the experiments shown in Fig. 5: Agilent E4428C signal generator, Mini-circuit ZAPD-30-S+ power splitter, Mini-circuit ZFBT-4R2GW bias tee, and Tektronix MDO3014 oscilloscope. The probing to the samples was conducted using a Technoprobe TP40-GSG-200-A GSG probe.

The following are the list of components used in the experiments shown in Fig. 6: Agilent E4428C signal generator, Digi-key D3C0802S circulator, mini-circuit ZHL-2-S+ amplifier, Pasternack PE2242-30 coupler as the coupler1, Pasternack PE2242-10 as the coupler2, Pasternack PE8013 power meter, Mini-circuit ZFBT-4R2GW bias tee, Agilent E5061B vector network analyzer, and Keithley 2000 and 2700 multimeters. The probing to the samples was conducted using a Technoprobe TP40-GSG-200-A GSG probe.

## Supplementary Materials

### This PDF file includes:

Figs. S1 to S26 Tables S1 to S14 Texts S1 to S11 References
