# Supplementary material for: Polymeric microwave rectifiers enabled by monolayer-thick ionized donors
Source: Sci Adv. 2025 Sep 19;11(38):eadv9952. doi: 10.1126/sciadv.adv9952 (PMC12448070; doi:10.1126/sciadv.adv9952)
Supplement: Supplementary file 1 — Figs. S1 to S26 Tables S1 to S14 Texts S1 to S11 References [file sciadv.adv9952_sm.pdf]

Science Advances
AAAS

Supplementary Materials for

Polymeric microwave rectifiers enabled by monolayer-thick ionized donors

Nobutaka Osakabe *et al.*

Corresponding author: Jun Takeya, takeya@k.u-tokyo.ac.jp; Yu Yamashita, yamashita.yu@nims.go.jp

Sci. Adv. **11**, eadv9952 (2025)
DOI: 10.1126/sciadv.adv9952

This PDF file includes:

Figs. S1 to S26
Tables S1 to S14
Texts S1 to S11
References

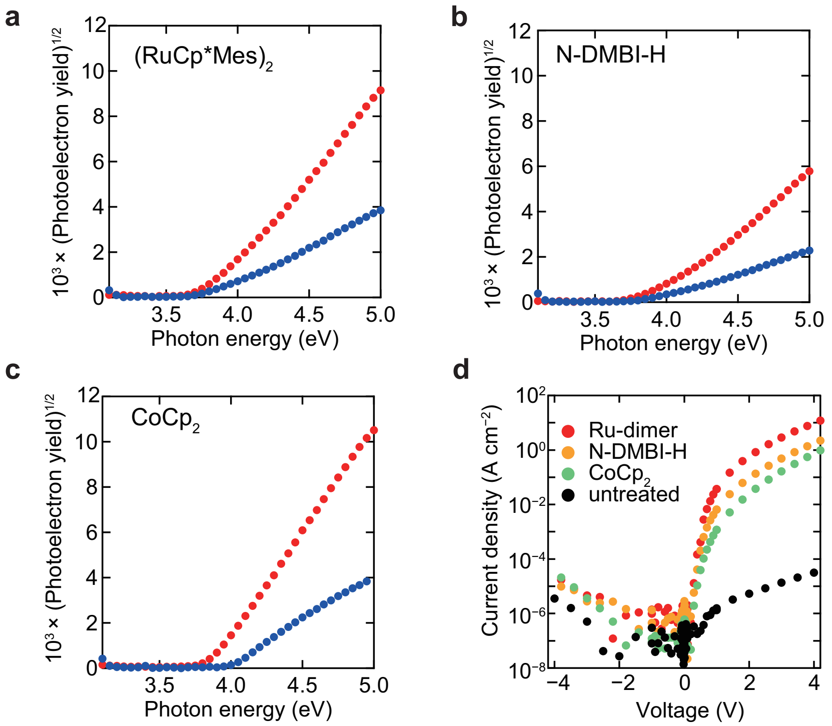

Figure S1: **Comparison of effects of donor molecules on work functions and injection properties.** PYS spectra obtained in vacuum and air for Au electrodes treated with (A) $(RuCp^*Mes)_2$, (B) N-DMBI-H, and (C) $CoCp_2$. (D) Diode properties measured in air for devices with treatments using the donor molecules.

Table S1: **Work functions of treated gold electrodes (eV).**

	Vacuum	Air
$(RuCp^*Mes)_2$	3.7	3.7
N-DMBI-H	3.8	3.8
$CoCp_2$	3.9	4.0
PEI	4.0	4.1
PFN-Br	4.3	4.1
untreated	4.8	-

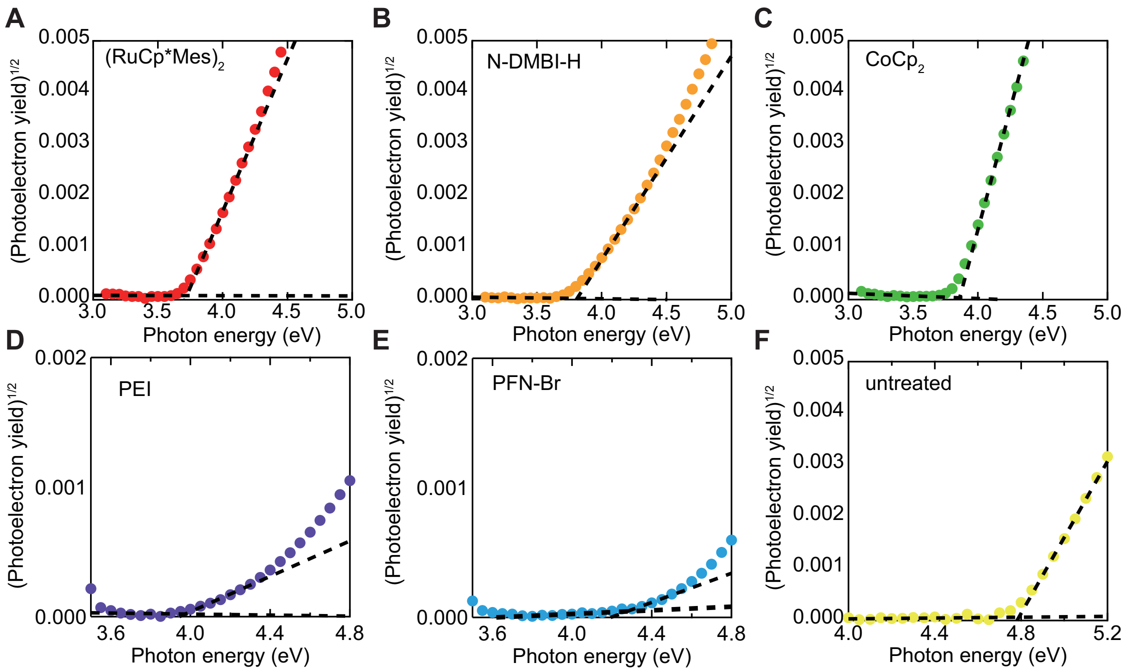

Figure S2: **PYS spectrum of gold electrodes treated under vacuum.** (A) $(RuCp^*Mes)_2$, (B) N-DMBI-H, (C) $CoCp_2$, (D) PEI, (E) PFN-Br, (F) none.

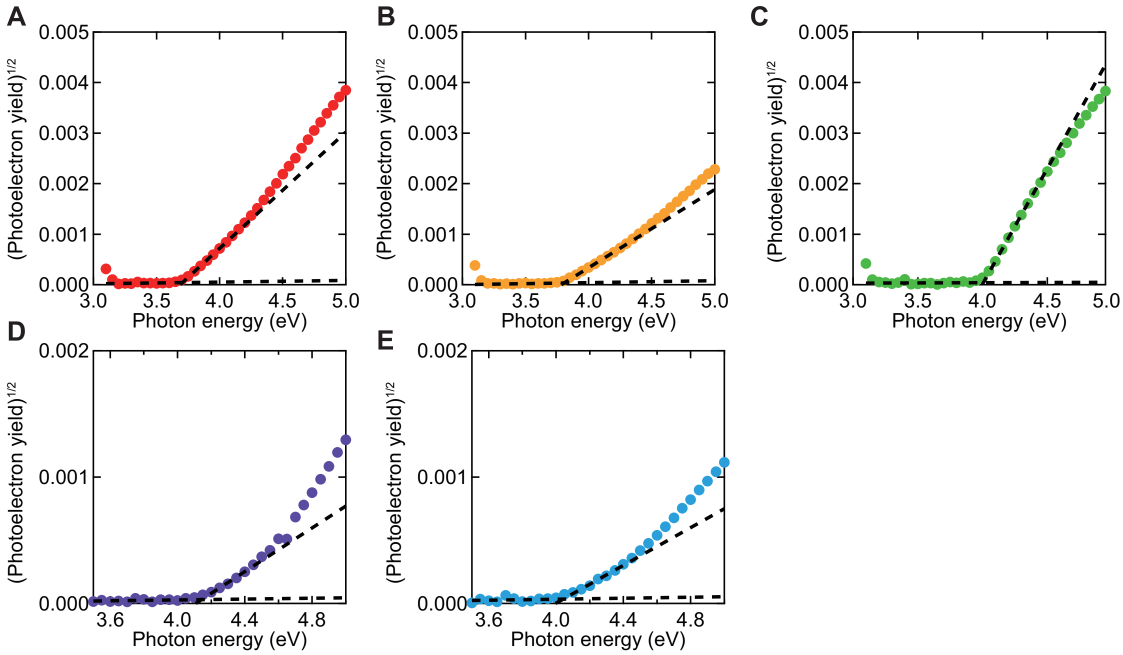

Figure S3: **PYS spectrum of Au treated in air.** (A) $(RuCp^*Mes)_2$, (B) N-DMBI-H, (C) $CoCp_2$, (D) PEI, (E) PFN-Br.

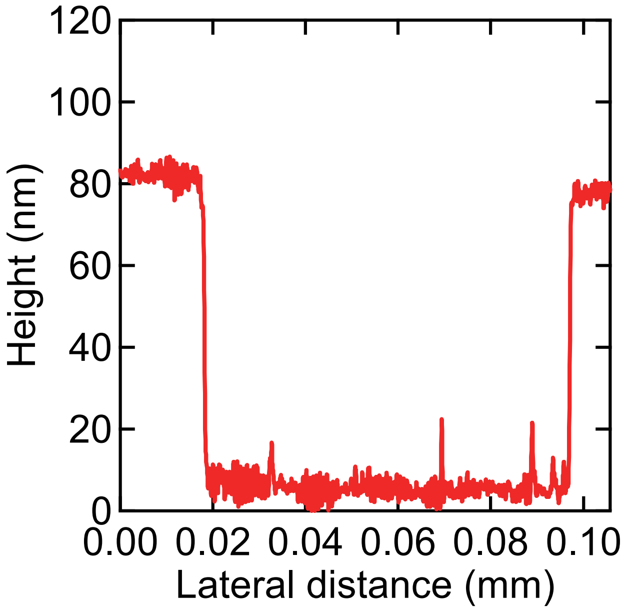

Figure S4: **Stylus profile of P(NDIOD-T2) coated using a 1 wt% solution.**

Supplementary Text 1. Analysis of XPS spectra of the surface treated electrodes

XPS survey spectra of the surface treated gold electrodes are shown in Fig. S5-S6. Fittings of narrow spectra are shown in Fig. S7-S13. The fitting results are summarized in Table S2-S13, where the relative peak area is the ratio of the peak area to the sum of the areas of the Au 4f peaks. The resulting atomic compositions are shown in Table S9. In this calculation, peak areas and relative sensitivity factors (RSFs) for each peak were employed, where RSFs suitable for our KRATOS ULTRA 2 setup were employed (C 1s: 0.28, N 1s: 0.48, O 1s: 0.78, Na 1s: 1.69, Br 3d: 1.06, Co 2p:2.39, Au 4f: 6.25, Ru 3d: 4.27). For Ru 3d peaks, $3d_{5/2}$ peak is observed but $3d_{3/2}$ is overlapping with C 1s. For this reason, known peak intensity ratio between them are assumed to calculate the atomic composition of Ru.

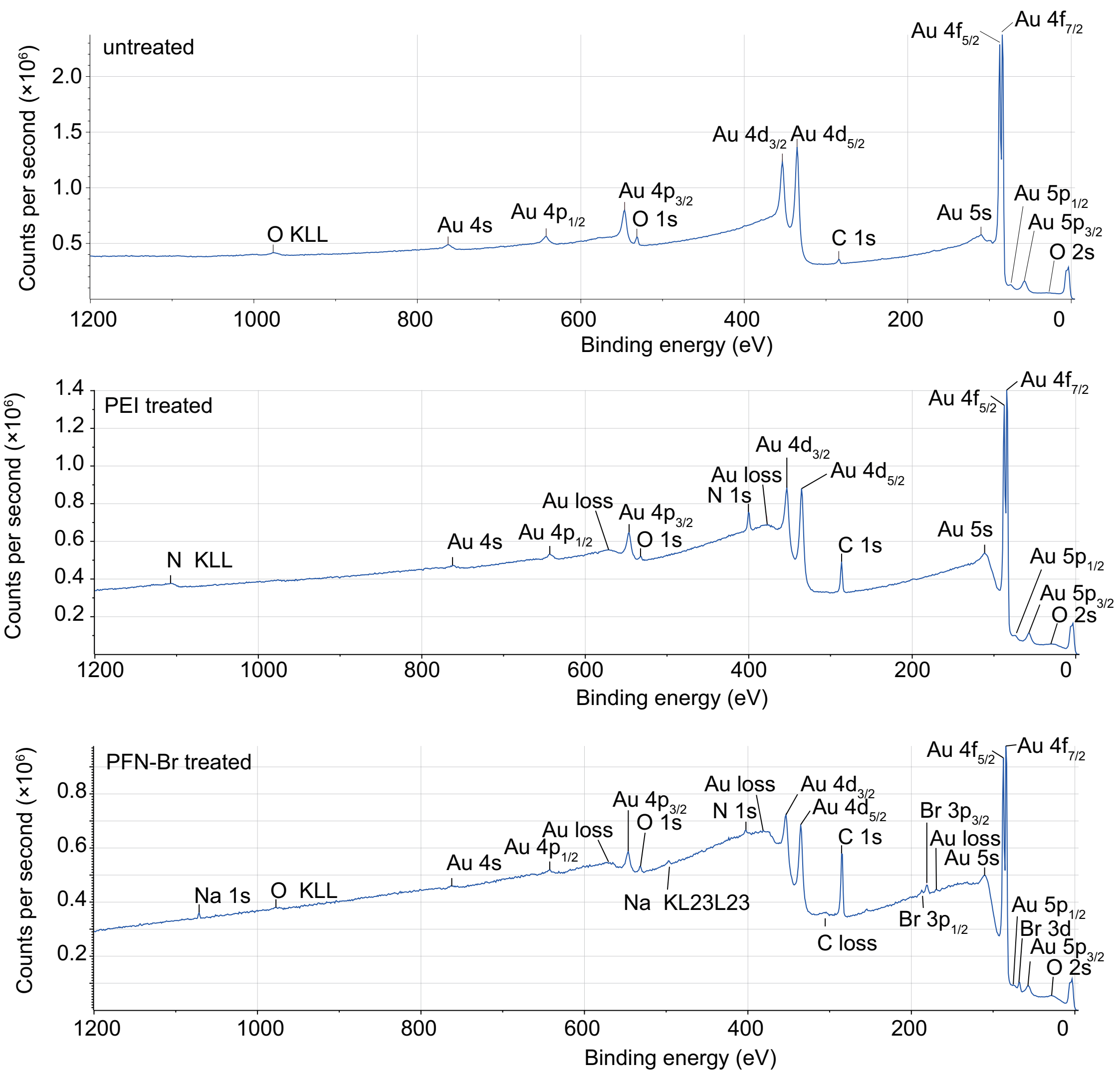

Figure S5: **XPS survey spectra of the untreated or treated gold electrodes.** Names of the interlayers employed are denoted.

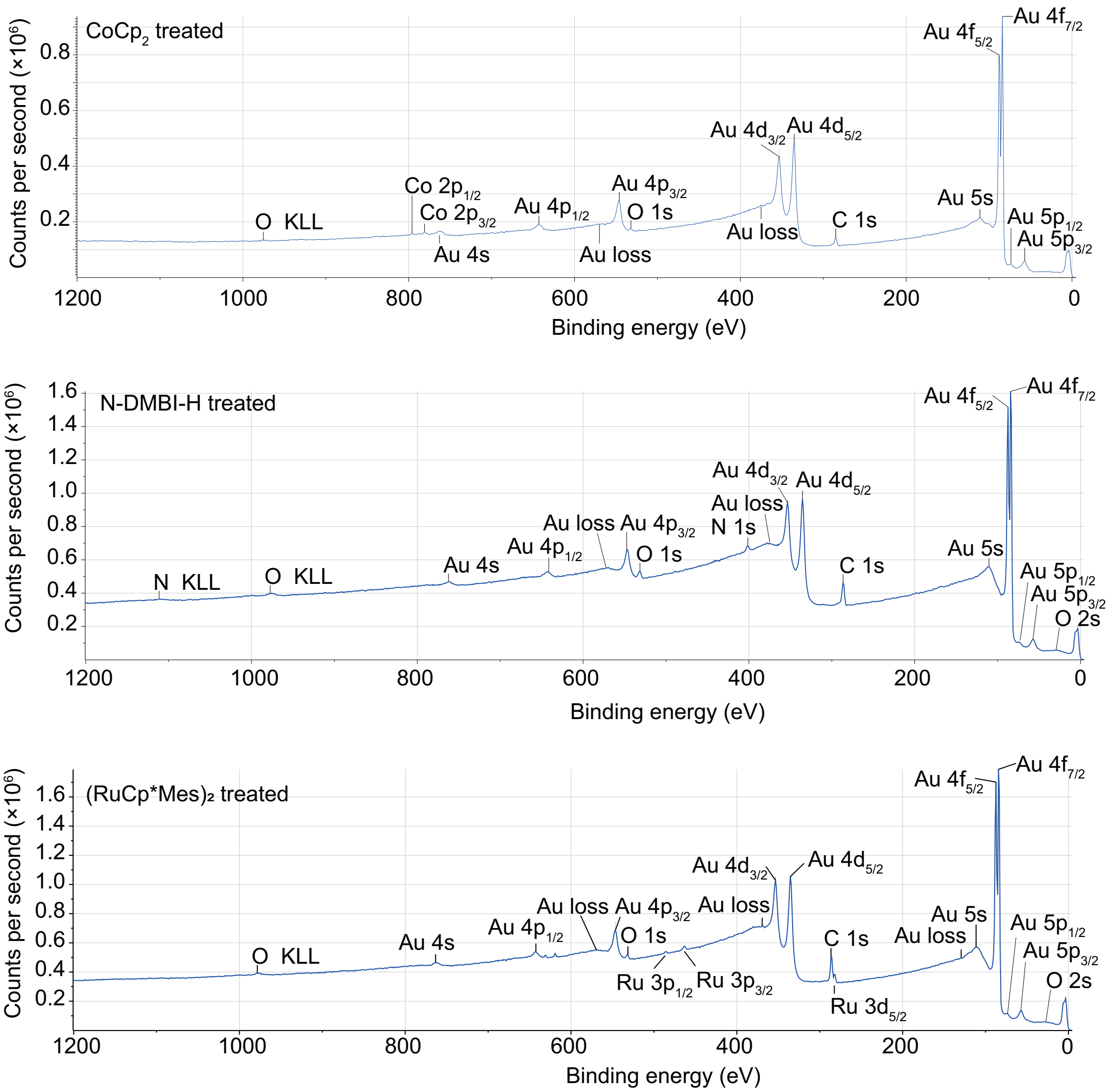

Figure S6: **XPS spectra in wide regions of the gold electrodes treated with n-type dopants.** Names of the donor molecules employed are denoted. The tiny peaks at 617 eV and 628 eV may originate from trace amount (less than 0.1 %) of iodide that may be impurities of the materials employed.

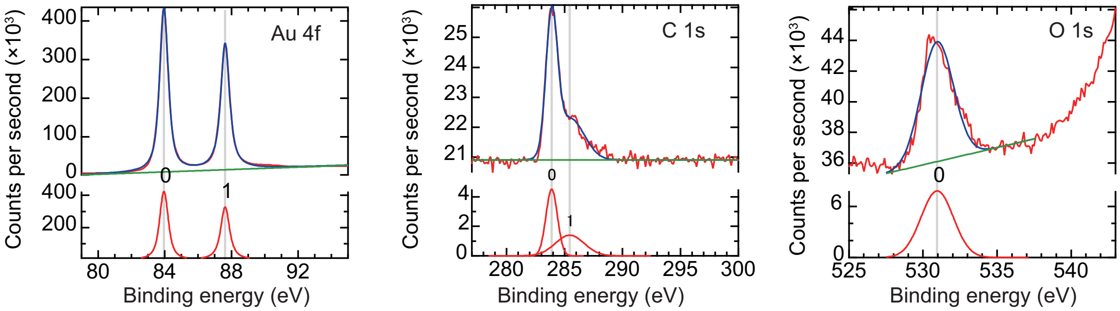

Figure S7: **XPS spectra of the untreated gold electrodes.**

Table S2: **XPS peak fittings for the untreated gold electrode.**

peak	binding energy (eV)	FWHM (eV)	relative peak area (%)
Au 4f (0)	83.949 ± 0.001	0.620 ± 0.004	54.5 ± 0.4
Au 4f (1)	87.624 ± 0.002	0.636 ± 0.006	45.5 ± 0.4
C 1s (0)	283.891 ± 0.008	1.16 ± 0.03	0.83 ± 0.04
C 1s (1)	285.4 ± 0.1	2.9 ± 0.2	0.65 ± 0.05
O 1s (0)	530.95 ± 0.03	2.56 ± 0.08	3.2 ± 0.1

To evaluate neutral and cationic nitrogen atoms in the PEI layer, the peak position of cationic atoms was assumed to 1.0 eV higher than the neutral one. Note that separation between these peaks is typically more than 1.0 eV. Based on this assumption, the ratio of cationic nitrogen atoms to the total one was estimated to be 17%.

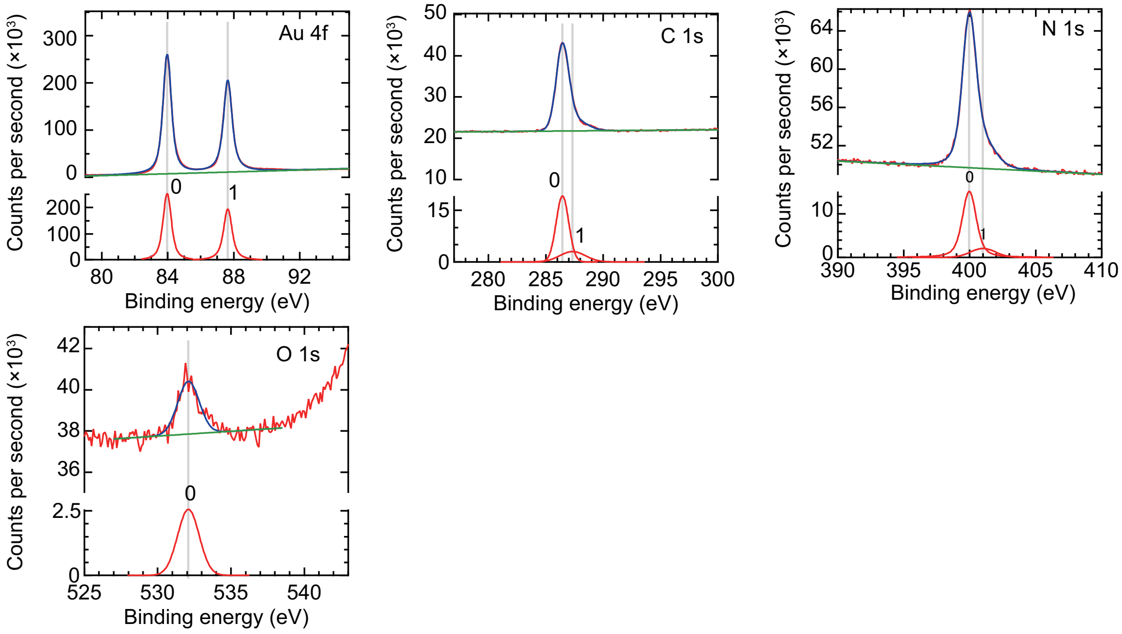

Figure S8: **XPS spectra of the PEI-treated gold electrodes.**

Table S3: **XPS peak fittings for the PEI-treated gold electrode.**

peak	binding energy (eV)	FWHM (eV)	relative peak area (%)
Au 4f (0)	83.966 ± 0.001	0.609 ± 0.004	54.7 ± 0.4
Au 4f (1)	87.640 ± 0.002	0.623 ± 0.006	45.3 ± 0.4
C 1s (0)	286.449 ± 0.003	1.28 ± 0.01	6.8 ± 0.1
C 1s (1)	287.33 ± 0.08	2.62 ± 0.08	2.2 ± 0.2
N 1s (0)	399.962 ± 0.004	1.257 ± 0.009	6.31 ± 0.09
N 1s (1)	401.0 ± -	2.23 ± 0.08	1.27 ± 0.06
O 1s (0)	532.10 ± 0.04	1.73 ± 0.09	1.2 ± 0.1

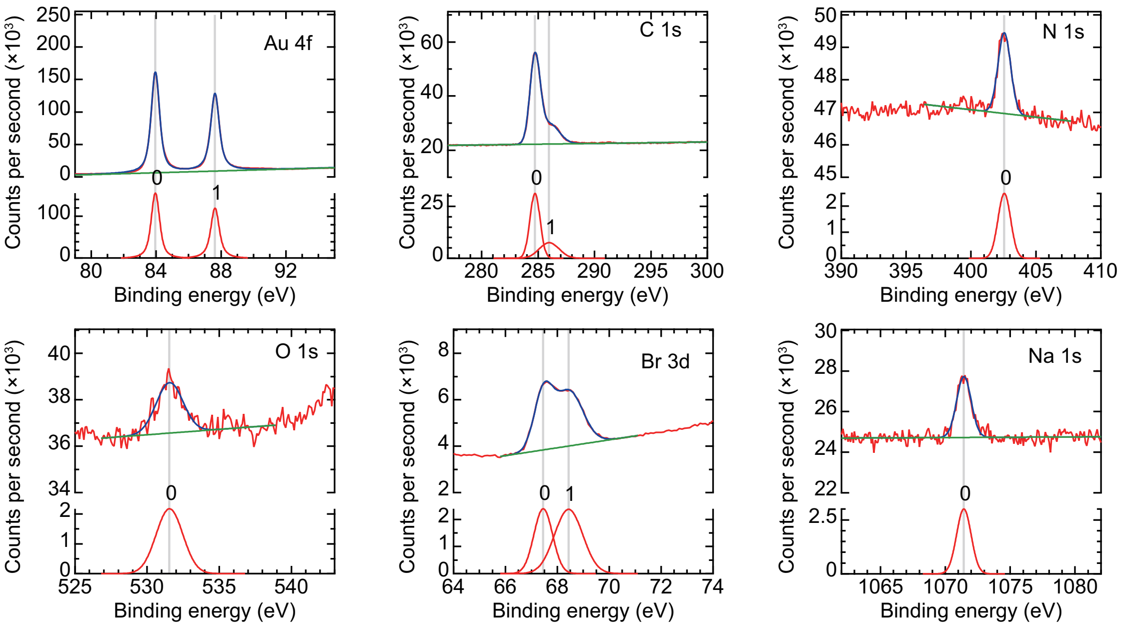

Figure S9: **XPS spectra of the PFN-Br treated gold electrodes.**

Table S4: **XPS peak fittings for the PFN-Br-treated gold electrode.**

peak	binding energy (eV)	FWHM (eV)	relative peak area (%)
Au 4f (0)	83.953 ± 0.001	0.617 ± 0.004	54.7 ± 0.4
Au 4f (1)	87.627 ± 0.002	0.629 ± 0.006	45.3 ± 0.4
C 1s (0)	284.725 ± 0.003	1.053 ± 0.009	14.7 ± 0.3
C 1s (1)	285.98 ± 0.05	2.06 ± 0.08	6.9 ± 0.3
N 1s (0)	402.57 ± 0.02	1.14 ± 0.04	1.27 ± 0.04
O 1s (0)	531.55 ± 0.05	2.2 ± 0.1	2.11 ± 0.13
Br 3d (0)	67.458 ± 0.009	0.84 ± 0.02	0.90 ± 0.04
Br 3d (1)	68.43 ± 0.02	1.28 ± 0.03	1.35 ± 0.04
Na 1s (0)	1071.43 ± 0.02	1.31 ± 0.05	1.76 ± 0.06

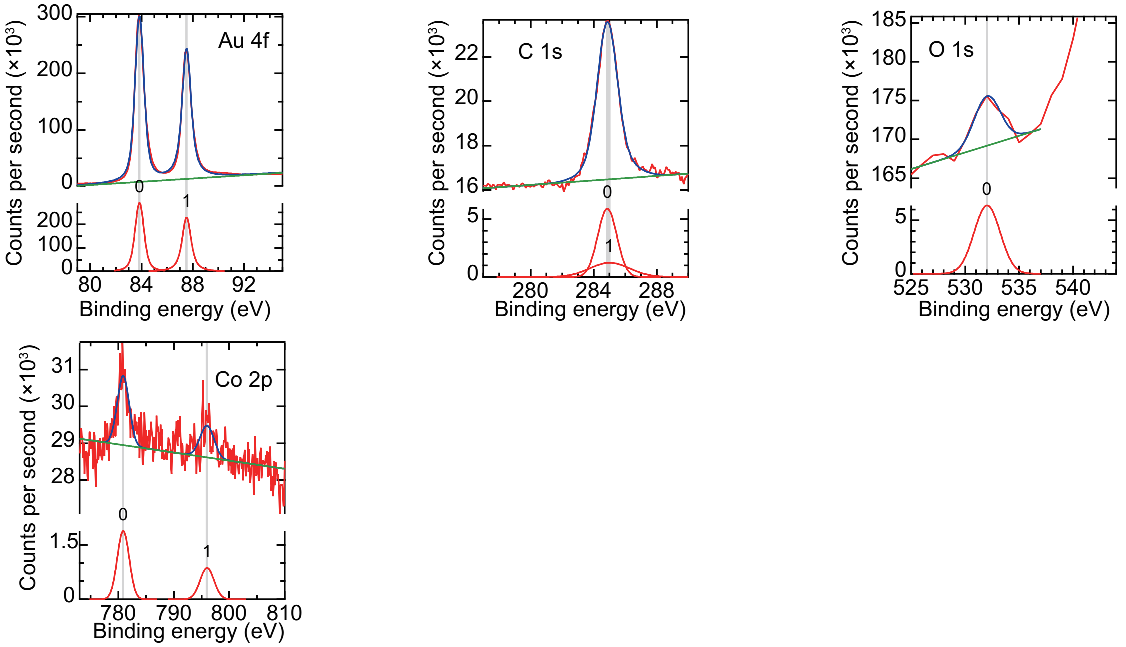

Figure S10: **XPS spectra of the $CoCp_2$-treated gold electrodes.**

Table S5: **XPS peak fittings for the $CoCp_2$-treated gold electrode.**

peak	binding energy (eV)	FWHM (eV)	relative peak area (%)
Au 4f (0)	83.844 ± 0.003	0.83 ± 0.01	53.4 ± 0.9
Au 4f (1)	87.519 ± 0.004	0.84 ± 0.02	46.6 ± 0.9
C 1s (0)	284.86 ± 0.01	1.42 ± 0.05	1.5 ± 0.2
C 1s (1)	285.01 ± 0.09	3.0 ± 0.4	0.7 ± 0.1
O 1s (0)	532.0 ± 0.2	2.8 ± 0.4	3.3 ± 0.5
Co 2p (0)	780.87 ± 0.08	2.5 ± 0.2	0.87 ± 0.06
Co 2p (1)	796.0 ± 0.2	2.9 ± 0.4	0.46 ± 0.07

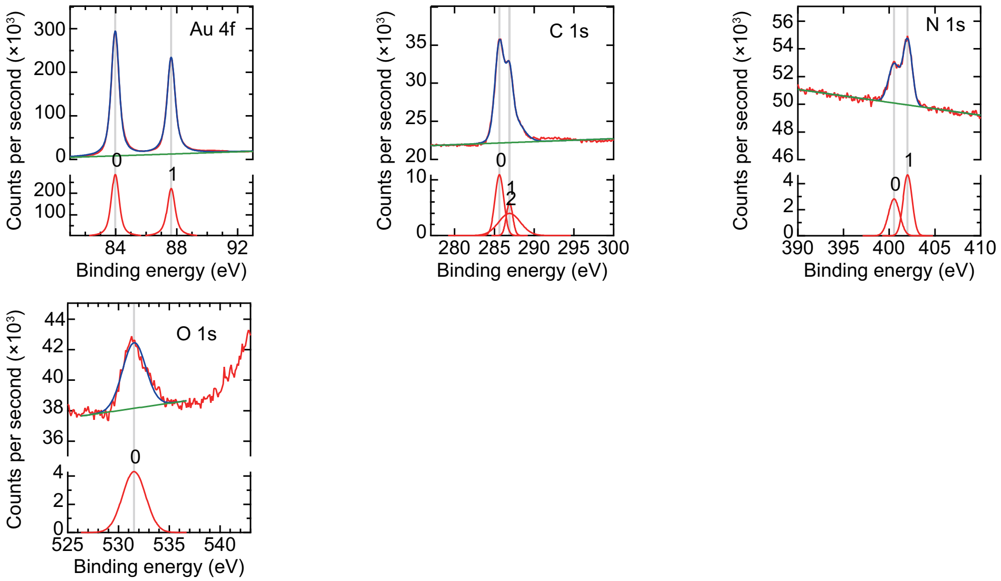

Figure S11: **XPS spectra of the N-DMBI-H-treated gold electrodes.**

Table S6: **XPS peak fittings for the N-DMBI-H-treated gold electrode.**

peak	binding energy (eV)	FWHM (eV)	relative peak area (%)
Au 4f (0)	83.964 ± 0.001	0.616 ± 0.004	54.6 ± 0.4
Au 4f (1)	87.639 ± 0.002	0.624 ± 0.006	45.4 ± 0.4
C 1s (0)	285.63 ± 0.01	1.30 ± 0.03	3.5 ± 0.2
C 1s (1)	286.90 ± 0.02	0.95 ± 0.04	1.3 ± 0.1
C 1s (2)	286.90 ± 0.08	3.2 ± 0.1	3.1 ± 0.2
N 1s (0)	400.53 ± 0.03	1.44 ± 0.07	0.98 ± 0.05
N 1s (1)	402.01 ± 0.02	1.16 ± 0.03	1.31 ± 0.05
O 1s (0)	531.54 ± 0.03	2.68 ± 0.09	2.8 ± 0.1

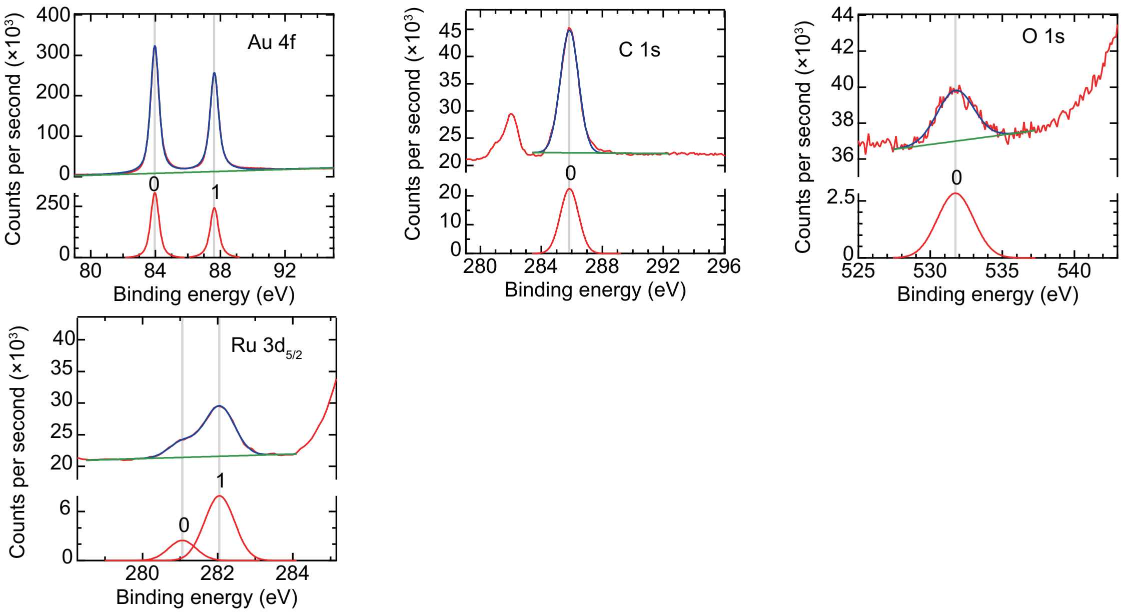

Figure S12: **XPS spectra of the (RuCp*Mes)$_2$-treated gold electrodes.**

Table S7: **XPS peak fittings for the (RuCp*Mes)$_2$-treated gold electrode.**

peak	binding energy (eV)	FWHM (eV)	relative peak area (%)
Au 4f (0)	83.959 ± 0.001	0.617 ± 0.004	54.6 ± 0.4
Au 4f (1)	87.635 ± 0.002	0.625 ± 0.006	45.4 ± 0.4
C 1s (0)	285.847 ± 0.005	1.41 ± 0.01	7.0 ± 0.1
O 1s (0)	531.74 ± 0.04	3.0 ± 0.1	1.8 ± 0.1
Ru 3d$_{5/2}$ (0)	281.06 ± 0.02	0.86 ± 0.04	0.47 ± 0.03
Ru 3d$_{5/2}$ (1)	282.048 ± 0.008	0.94 ± 0.02	1.63 ± 0.03

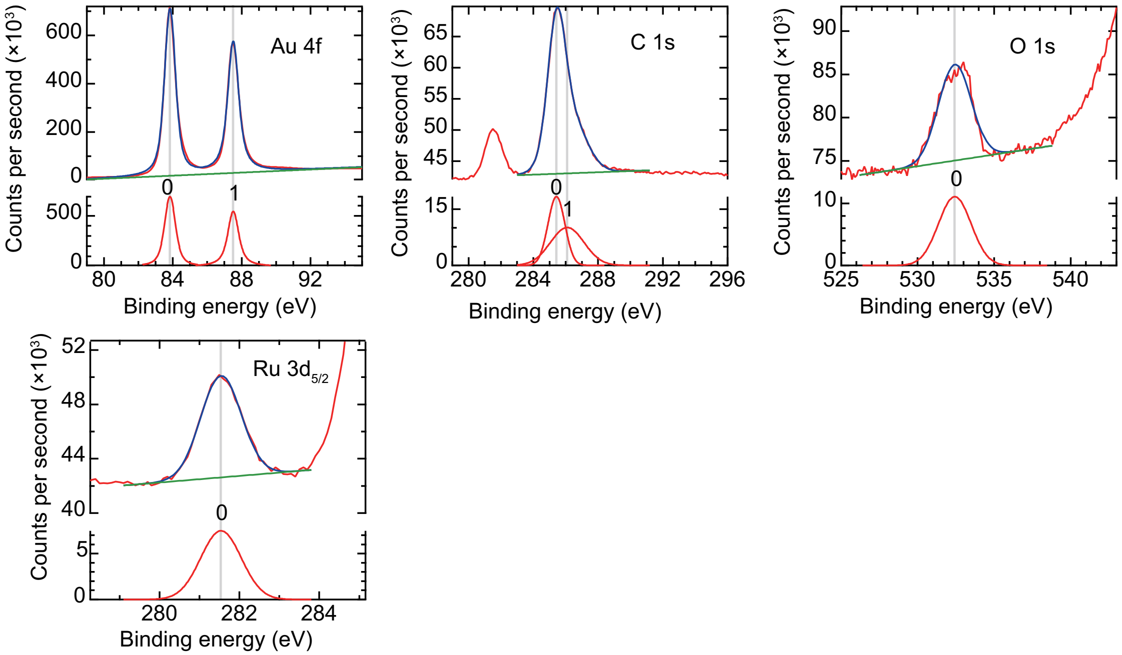

Figure S13: **XPS spectra of the $(RuCp^*Mes)_2$-treated gold electrodes after *o*DCB washing.**

Table S8: **XPS peak fittings for the $(RuCp^*Mes)_2$-treated gold electrodes after *o*DCB washing.**

peak	binding energy (eV)	FWHM (eV)	relative peak area (%)
Au 4f (0)	83.835 ± 0.003	0.761 ± 0.008	0.538 ± 0.008
Au 4f (1)	87.511 ± 0.003	593106 ± 9123.4	0.462 ± 0.007
C 1s (0)	285.428 ± 0.005	1.23 ± 0.02	0.0187 ± 0.0007
C 1s (1)	286.10 ± 0.04	2.44 ± 0.04	0.0205 ± 0.0009
O 1s (0)	532.43 ± 0.02	2.51 ± 0.06	0.0231 ± 0.0006
Ru $3d_{5/2}$ (0)	281.540 ± 0.006	1.19 ± 0.02	0.0074 ± 0.0001

Table S9: **Atomic compositions of untreated and treated gold electrodes (%)**

employed treatment	Au	C	N	O	Na	Co	Br	Ru
untreated	63.2	20.8	-	16.1	-	-	-	-
PEI	24.8	49.8	23.0	2.4	-	-	-	-
PFN-Br	15.8	75.8	2.6	2.7	1.0	-	2.1	-
$CoCp_2$	62.9	30.8	-	4.2	-	2.2	-	-
N-DMBI-H	30.4	53.6	9.1	6.9	-	-	-	-
$(RuCp^*Mes)_2$	41.0	50.8	-	6.1	-	-	-	2.1
$(RuCp^*Mes)_2$, *o*DCB washed	50.9	38.7	-	9.4	-	-	-	0.9

Supplementary Text 2. Evaluation of overlayer thickness based on XPS spectra

Signals from depth z in the XPS measurements are attenuated by a factor of $\exp(-z/\lambda)$, where the inelastic mean free path (IMFP) of photoelectrons can be employed as λ. When there are overlayer on the target substrate with different IMFPs (λ_{ov} and λ_{sub}), the signal from the substrate will be attenuated by $\exp(-t/\lambda_{ov})$ compared to the case without the overlayer, where t is the thickness of the overlayer (*51*). In this situation, the signals from depth z will be attenuated by $\exp(-z/\lambda_{ov})$ for the signals from the overlayer and $\exp(-t/\lambda_{ov})\exp(-(z-t)/\lambda_{sub})$ for the signals from the substrate. We constructed model structures based on the overlayers of organic molecular materials and Au with different IMFPs for photoelectrons (Fig. S14A). An IMFP of 17.3 Å was employed for Au (*52*). For organic or metal-organic molecular materials, the known IMFPs for kaptone (32.5 Å) and polystyrene (37.2 Å) (*53*) were employed. The organic molecular layer was modelled to have 1 g cm^{-3} density of carbon atoms. The density of Au atoms was based on its crystal structure. Signals from depth z were calculated for varying z at a step of 0.1 Å and summed over the entire depth to calculate the expected Au composition that will be evaluated by XPS measurements. The model structures included varying thicknesses of the overlayer and Au with a total thickness of 50 nm. The calculated dependence of the expected Au contribution to XPS signals on the overlayer thickness is plotted in Fig. S14B. Using this relationship, the thicknesses of the molecular layers on the treated Au electrodes were calculated, as shown in the Fig. 2C. Here, the averages and errors were calculated using IMFPs of 32.5 Å and 37.2 Å for the molecular layer.

In the above method, while the thickness of the molecular layer can be estimated from XPS measurements, the evaluated thickness is expected to include one for the carbon layer that is usually adsorbed on the surface upon air exposure. We also constructed models to calculate the atomic composition of Ru (Fig. S15). The model structure is composed of a monolayer of RuCp*Mes$^+$ on an Au layer. To calculate the density of the RuCp*Mes$^+$ in the monolayer, cylindrical structures with the same radius and height as the RuCp*Mes$^+$ molecule were assumed to have a dense hexagonal packing, which yielded 1.3×10^{14} cm^{-2}. Another model employing an additional carbon overlayer on the Au surface was developed. The carbon overlayer thickness was fixed at the value evaluated for the untreated Au electrode in our XPS measurements (9.2 Å). The calculation method used in Fig. S14 was employed for these models, and the results are listed in Table S10. The Ru atomic compositions were similar for our model calculations and the experiment that employed

the $(RuCp^*Mes)_2$-treatment and *o*DCB washing. When the carbon overlayer was employed in the model calculation, the atomic compositions of Au and C were also comparable to the experimental ones. The remaining difference between the model calculation and experiment may arise from the oxygen atoms that were not considered in our model calculations. These results support that our method using $(RuCp^*Mes)_2$-treatment and *o*DCB washing leaves a $RuCp^*Mes^+$ layer with a thickness close to a monolayer on the gold electrodes.

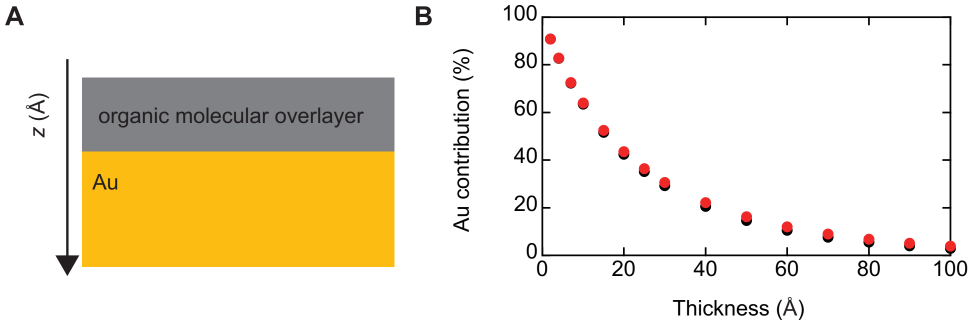

Figure S14: **Evaluation of overlayer thickness based on XPS spectra** (A) Illustration of a model structure. (B) Relationship between the Au contribution to XPS spectra and the overlayer thickness. IMFPs of the overlayer were 32.5 Å for the black plot and 37.2 Å for the red plot.

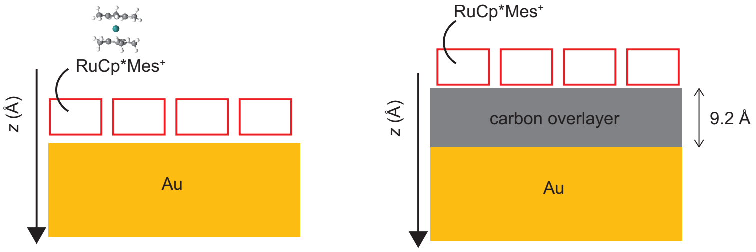

Figure S15: **Model structures for $(RuCp^*Mes)_2$-treated Au electrodes.** Models without (left) and with (right) an additional carbon overlayer are illustrated.

Table S10: **Calculated atomic compositions of (RuCp*Mes)$_2$-treated gold electrodes (%).**

	Au	C	Ru	O
model w/o carbon layer	81.7	17.1	1.4	-
model w/ carbon layer	55.3	43.6	1.0	-
experimental (*o*DCB washed)	50.9	38.7	0.9	9.4

Supplementary Text 3. Analysis of XPS spectra of the electrode/semiconductor structures

In our study, the electrode and semiconductor layers were analyzed using Ar-ion gun etching and by comparing samples with varying semiconductor thicknesses. For the etching experiment shown in Fig. 3, more detailed time-dependent data are presented in Fig. S16. This experiment was performed using a 2 keV Ar-ion gun with a raster size of 1.5 mm. To detect the weak Ru signal, a large measurement area of 300× 700 μm (Slot mode) was employed. For more quantitative analysis, it is preferable to use a measurement area sufficiently smaller than the raster size. However, due to limitations in signal strength, we instead supplemented the analysis with a complementary experiment using samples with different semiconductor thicknesses, as described below.

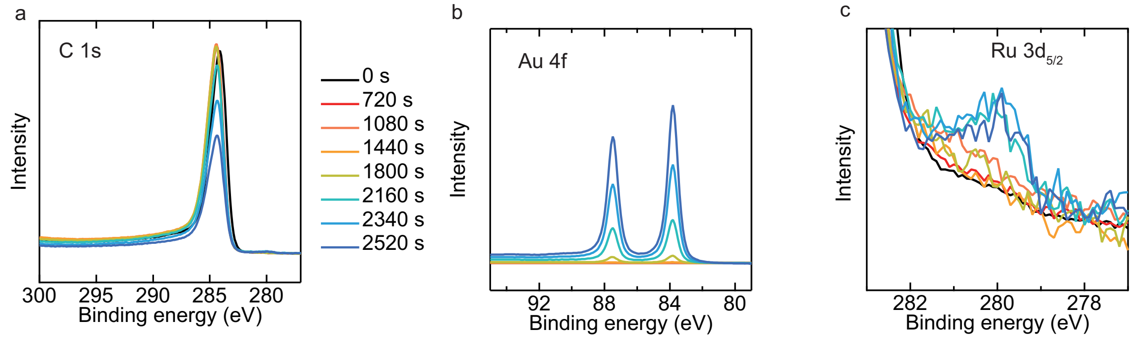

Figure S16: **XPS spectra of samples after Ar-ion gun etching.** (a) C 1s, (b) Au 4f, (c) Ru $3d_{5/2}$ regions of the samples with $(RuCp^*Mes)_2$-treated gold electrodes with spin-coated P(NDIOD-T2). The employed etching times are denoted as legends.

For the analysis using samples with varying semiconductor thickness, P(NDIOD-T2) solutions with varying concentrations were spin-coated on Au electrodes with and without the $(RuCp^*Mes)_2$-treatment. The resulting samples were analyzed using XPS. The results for the sample without the treatment are summarized in Fig. S17 and Table S11, and one with the treatment are summarized in Fig. S18 and Table . Ru 3d peak was observed only when 0.2wt% P(NDIOD-T2) solution was coated on the treated Au. This supports that RuCp*Mes was confined close to the Au interface. In this measurement, the ratio of atomic composition Ru/Au was 1.8%. This value is consistent with the case without P(NDIOD-T2) layer after *o*DCB washing (Fig. S13 and Table S8), whose Ru/Au ratio was 1.8%. This supports that comparable amount of RuCp*Mes was present around the Au interface even after P(NDIOD-T2) was coated on the $(RuCp^*Mes)_2$-treated Au.

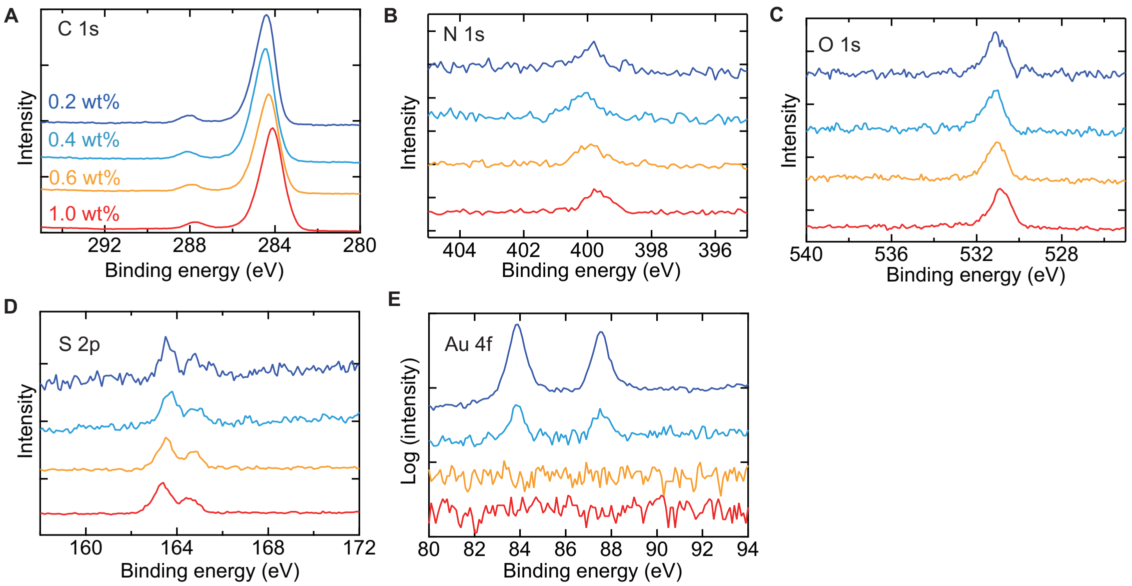

Figure S17: **XPS spectra of samples with P(NDIOD-T2) layers with varying thickness on untreated Au.** The concentrations of employed P(NDIOD-T2) solutions are denoted. (A) C 1s, (B) N 1s, (C) O 1s, (D) S 2p, (E) Au 4f regions. Offsets of spectra are modified to improve visibility.

Table S11: **Atomic compositions of samples with P(NDIOD-T2) layers with varying thickness on untreated Au (%).**

sample	C	N	O	S	Au
0.2wt%	86.8 ± 7.7	4.1 ± 0.2	5.0 ± 0.4	2.0 ± 0.2	2.13 ± 0.02
0.4wt%	91.1 ± 4.0	2.8 ± 0.2	4.7 ± 0.3	1.3 ± 0.1	0.08 ± 0.01
0.6wt%	90.4 ± 5.7	2.6 ± 0.2	4.7 ± 0.2	2.3 ± 0.2	- ± -
1.0wt%	90.4 ± 6.4	2.8 ± 0.2	5.2 ± 0.2	1.6 ± 0.1	- ± -

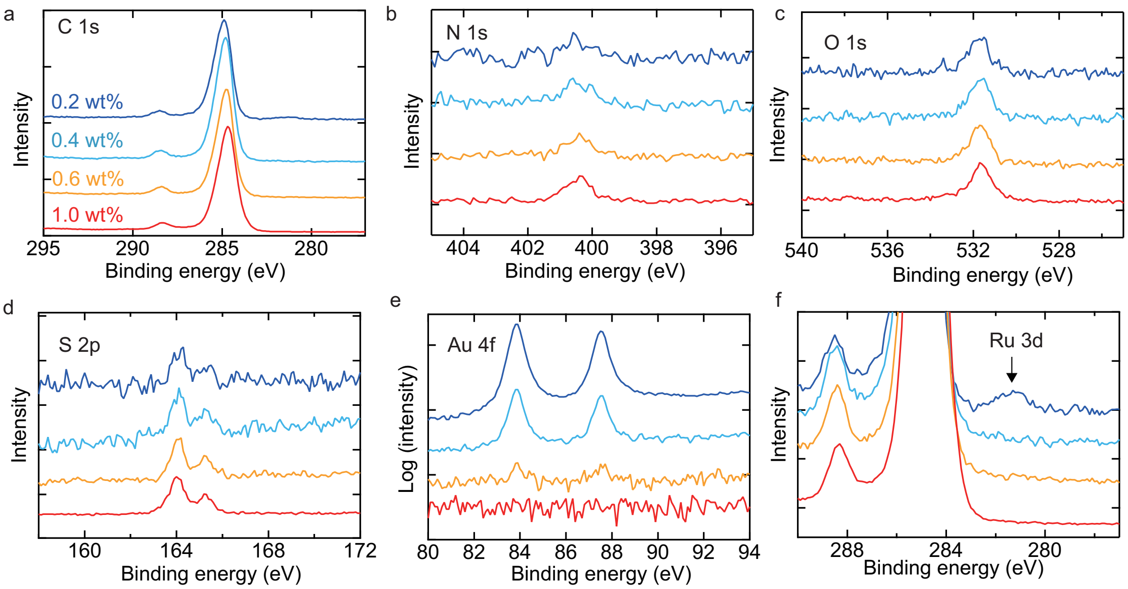

Figure S18: **XPS spectra of samples with P(NDIOD-T2) layers with varying thickness on ($RuCp^*Mes)_2$-treated Au.** The concentrations of employed P(NDIOD-T2) solutions are denoted. (A) C 1s, (B) N 1s, (C) O 1s, (D) S 2p, (E) Au 4f (F) Ru 3d regions. Offsets of spectra are modified to improve visibility.

Table S12: **Atomic compositions of samples with P(NDIOD-T2) layers with varying thickness on ($RuCp^*Mes)_2$-treated Au (%).**

sample	C	N	O	S	Ru	Au
0.2wt%	80.7 ± 7.6	3.67 ± 0.02	5.2 ± 0.5	1.5 ± 0.2	0.15 ± 0.01	8.71 ± 0.07
0.4wt%	88.7 ± 3.8	3.6 ± 0.5	4.9 ± 0.3	1.9 ± 0.2	- ± -	0.91 ± 0.02
0.6wt%	90.2 ± 2.6	2.6 ± 0.3	5.1 ± 0.3	2.2 ± 0.2	- ± -	0.028 ± 0.003
1.0wt%	88.9 ± 3.9	3.0 ± 0.2	5.8 ± 0.2	2.3 ± 0.1	- ± -	- ± -

Supplementary Text 4. Analysis of UPS spectra.

To determine the Fermi energy (E_F), the UPS spectrum of a clean gold surface was measured.

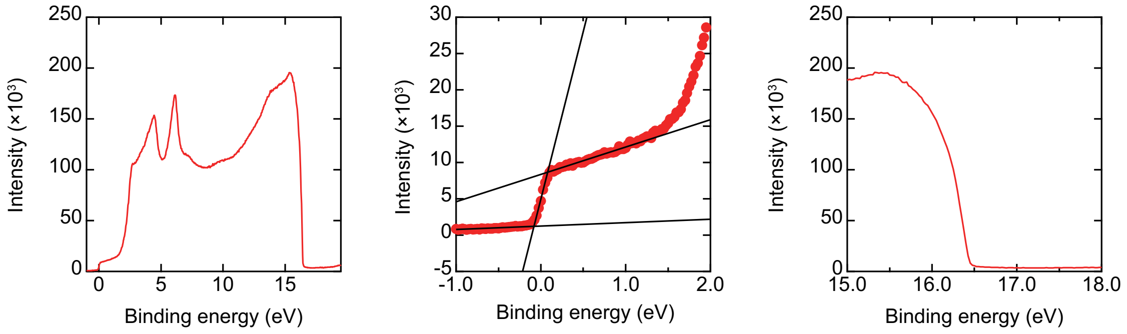

Figure S19: **UPS spectra of the cleaned gold electrode.**

This sample was fabricated by depositing 3 nm of Cr and 30 nm of Au. After loading the sample from air to the measurement chamber, Ar ion gun was employed to make a clean surface. The obtained UPS spectrum is shown in Fig. S19 whose origin is the E_F determined from this sample. The E_F was determined as the middle of the two intersections between linear fitting lines shown in the middle panel of Fig. S19.

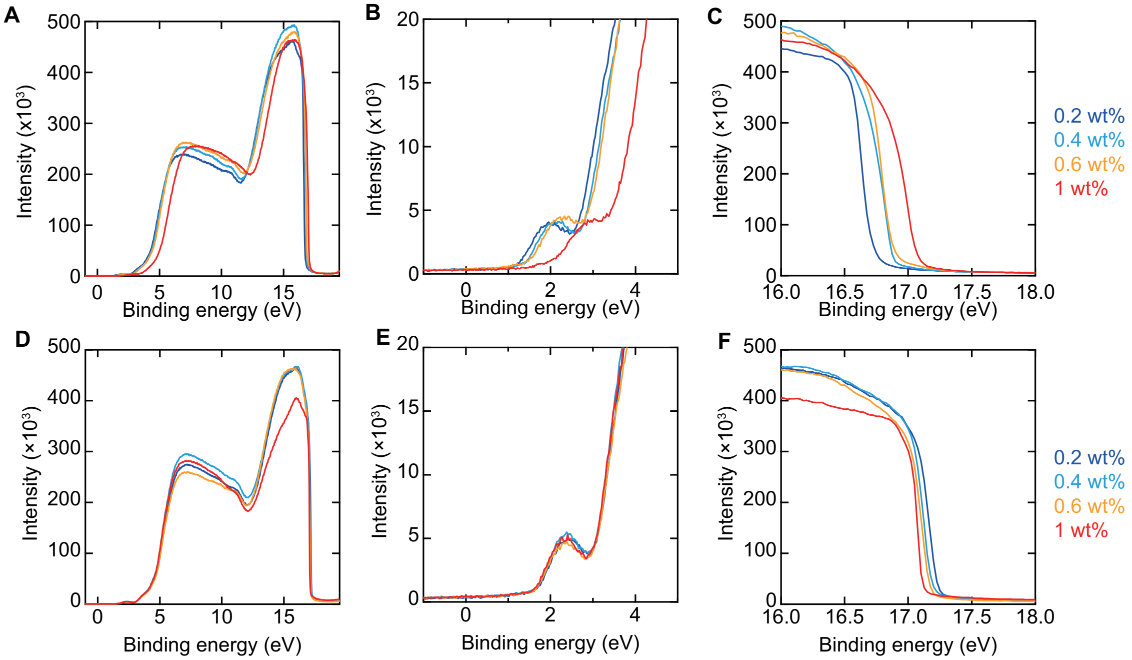

Figure S20: **UPS spectra of gold electrodes covered by P(NDIOD-T2) thin films.** (A-C) Samples with untreated gold electrodes. (D-F) Samples with $(RuCp^*Mes)_2$ treated gold electrodes. Concentrations of the employed P(NDIOD-T2) solutions are denoted. The origin of binding energy is the Fermi level that is determined by measuring an clean Au surface.

Supplementary Text 5. Maximum frequency predicted based on the mobility

Considering the transit time of charge carriers, attainable maximum operational frequency $f_{\max}$ depends on the applied bias to the diode and carrier mobility (μ) of the employed semiconductor in the vertical direction. $f_{\max}$ has been described in following equations (*41*).

$$f_{\max} = \mu(V_A - V_{d.c.})/L^2 \tag{S1}$$

$$f_{\max} = \frac{9\mu}{16\pi L_D^2 V_{d.c.}}[(-3V_{d.c.} + V_F)\sqrt{V_A^2 - (V_{d.c.} + V_F)^2} + (V_A^2 + 2V_{d.c.}^2)arccos(\frac{V_{d.c.} + V_F}{V_A})] \tag{S2}$$

V_A is the input voltage in the forward direction, $V_{d.c.}$ is the rectified DC voltage, L is the thickness of semiconductor, L_D is the thickness of depletion layer, V_F is the transition voltage. We employed μ of 0.1 cm^2 V^{-1} s^{-1}, V_A of 3 V, $V_{d.c.}$ of 1 V, V_F of 0.5 V and plotted $f_{\max}$ against the thickness (L for equation S1 and L_D for equation S2) in Fig. S21. From this plot, when thickness of semiconductor is below 100 nm, GHz operation is possible even for the mobility of 0.1 cm^2 V^{-1} s^{-1}.

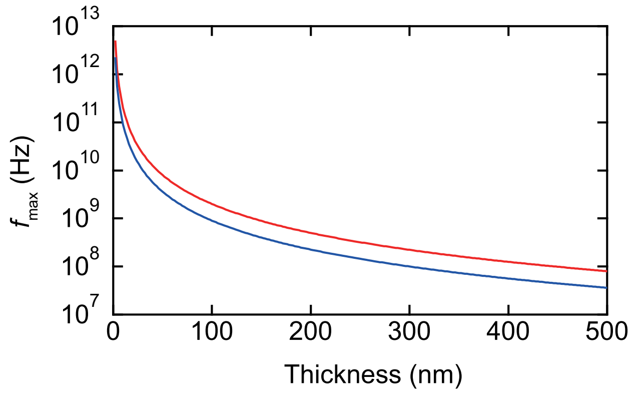

Figure S21: **Maximum operation frequency predicted based on the carrier mobility.** The red line is based on the equation (S1) and the blue line is based on the equation (S2).

Supplementary Text 6. Depletion layer thickness of the diodes

In Fig. 6, the thin P(NDIOD-T2) diode fabricated using 0.5wt% solution was employed. The depletion layer thickness for this condition was evaluated based on impedance measurements shown in Fig. S22. Here, Z_{im} was inversely proportional to the frequency. This suggests that following equations are valid regarding the depletion layer thickness t, whose approximation is applicable only at enough high frequencies.

$$Z_{\mathrm{im}} = 1/\omega C_{\mathrm{b}} \tag{S3}$$

$$C_{\mathrm{b}} = \epsilon S/t \tag{S4}$$

C_{b} is the depletion layer capacitance, S is the area of diode, ϵ is permittivity. Relative permittivity of P(NDIOD-T2) has been reported to be 3 (*54*). Based on this, the depletion layer thickness of the diode was calculated to be 35 nm.

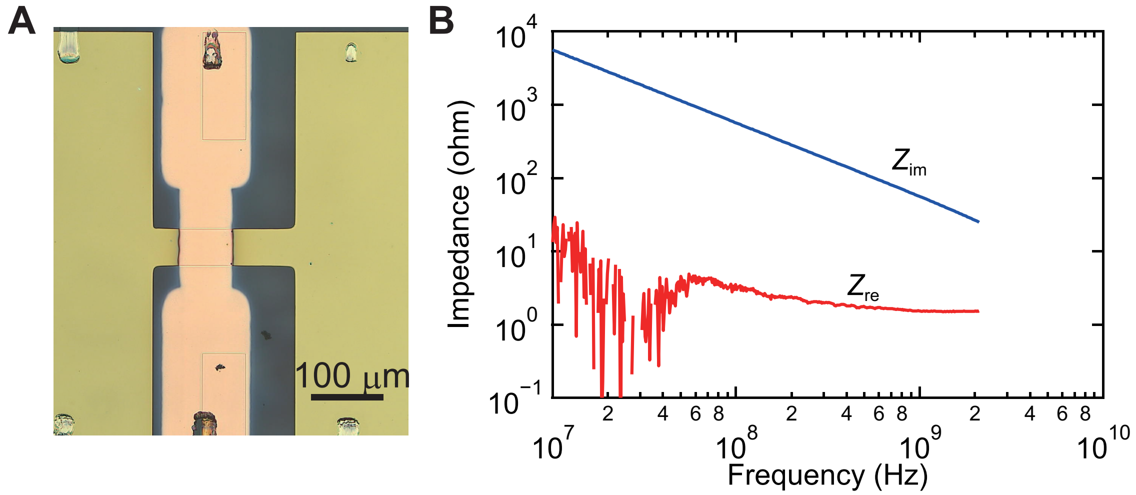

Figure S22: **Impedance analysis of diodes shown in Fig. 6.** (A) A microscopy image of the diode. (B) The measurement of the frequency dependent impedance of diode. The red line shows the real part of impedance and the blue line shows the imaginary one.

Supplementary Text 7. Reproducibility of diode property

The reproducibility of our diodes was evaluated under conditions designed to fabricate depletion layers with thicknesses of 80 nm and 35 nm, with 28 devices fabricated on a single chip. Compared to the diodes with 80-nm-thick depletion layers, those with 35-nm-thick depletion layers exhibited poorer reproducibility, with some devices showing short circuits. Nevertheless, more than half of the diodes exhibited a rectification ratio close to 10^3. The successful fabrication of thin diodes without short circuits during the evaporation of the top electrodes may be attributed to the high molecular weight of P(NDIOD-T2) (Mw: 202,261) and its dense out-of-plane π stacking structure.

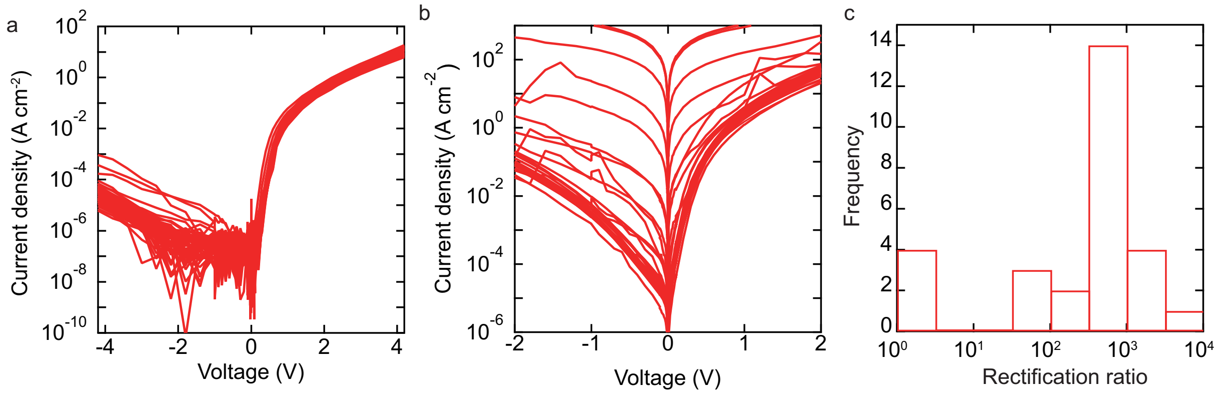

Figure S23: **Reproducibility of diodes.** (a) Performance of 56 diodes (on two chips) with 80-nm-thick depletion layers. (b) Performance of 28 diodes with 35-nm-thick depletion layers and (c) the histogram of their rectification ratio. The Area of the diodes are 3750 μm^2.

Supplementary Text 8. Stability against reverse bias

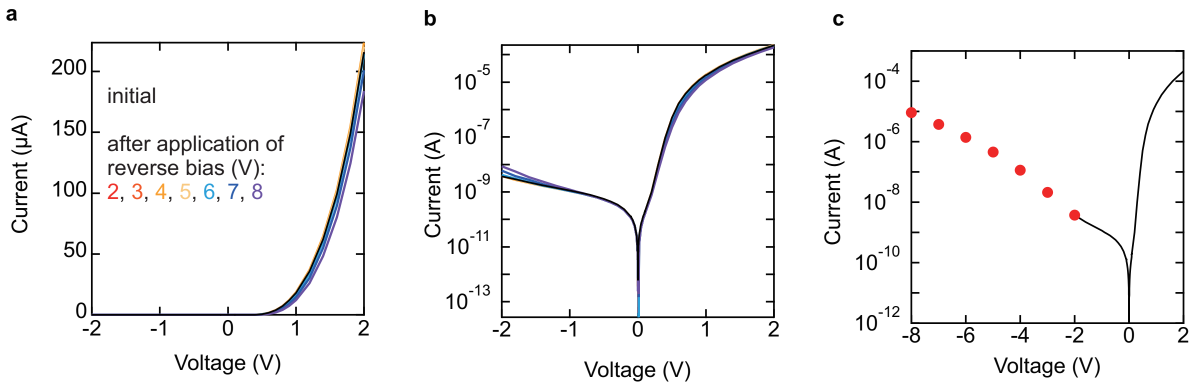

Figure S24: **Stability against reverse bias.** (a) Linear-scale and (b) logarithmic-scale current–voltage characteristics of a diode with a 35-nm-thick depletion layer after the application of large reverse biases. The indicated reverse biases were applied one minute prior to the measurement. (c) The measured current during the application of the reverse bias (red markers) together with the diode characteristics.

Supplementary Text 9. Calculation of rectification efficiency

The power conversion efficiencies of our diodes were calculated using the setup shown in Fig.6. By measuring the input power P_{in} and reflected power P_{re} using the signals from the coupler 1 and coupler 2, respectively, the effective input power to the diode was calculated as $P_{\mathrm{in,eff}} = P_{\mathrm{in}} - P_{\mathrm{re}}$. The output DC power was determined based on the load resistance R_{L} and output voltage V_{out}: $P_{\mathrm{out}} = V_{\mathrm{out}}^2/R_{\mathrm{L}}$. The AC-to-DC power conversion efficiency η and η_{int} were calculated as $P_{\mathrm{out}}/P_{\mathrm{in}}$ and $P_{\mathrm{out}}/P_{\mathrm{in,eff}}$. The actual obtained values are listed in Table S13.

The power conversion efficiencies of the diodes reported in the literatures were calculated from the available information. In most studies, the AC input voltages V_{in} and DC output voltages V_{out} were monitored using an oscilloscope, where the input signals were split into diodes and an oscilloscope with 50 Ω termination. In this case, the input power was calculated as $P_{\mathrm{in}} = V_{\mathrm{in}}^2/50$ in our calculation. This is the upper limit of power introduced to the diodes in such measurements. The output DC power was calculated based on the measured output voltages V_{out} and the load resistance R_{L} between the diodes and ground line. For instance, when 1 V is observed using an oscilloscope with an input impedance of 1 MΩ and there are no other load resistors, R_{L} is 1 MΩ and the output power P_{out} that is consumed by the system is 1 μW. The efficiency was determined by calculating $P_{\mathrm{out}}/P_{\mathrm{in}}$. The intrinsic performance of the devices may be higher than the efficiencies calculated in this method, where impedance matching and measurements with varying load resistance are necessary to assess the attainable efficiency with a diode. Note that even if ideal measurements are conducted to assess attainable efficiency with the devices, our diode is expected to show higher efficiency around 1 GHz compared to reported devices owing to our diode's capability to pass high currents at a low bias as shown in the next section.

Table S13: **The obtained data in our rectification efficiency measurements.**

P_{in}	R_{load} (Ω)	P_{re} (mW)	$P_{in,eff}$ (mW)	V_{out} (V)	P_{out} (mW)	η (%)	η_{int} (%)
67.7	10^6	28.7	39.1	1.87	0.0035	0.0052	0.0089
67.7	10^4	28.5	39.2	1.66	0.28	0.41	0.70
67.7	5000	28.4	39.3	1.54	0.47	0.70	1.2
67.7	3000	28.1	39.6	1.42	0.67	1.0	1.7
67.7	2000	27.8	39.9	1.32	0.87	1.3	2.2
67.7	1000	27.2	40.5	1.11	1.24	1.8	3.1
67.7	700	26.8	40.9	1.00	1.43	2.1	3.5
67.9	500	26.5	41.4	0.89	1.58	2.3	3.8
67.9	300	25.5	42.4	0.72	1.74	2.6	4.1
67.9	200	24.9	43.0	0.59	1.76	2.6	4.1
68.1	100	23.6	44.5	0.40	1.56	2.3	3.5
68.2	50	22.5	45.7	0.24	1.19	1.7	2.6
108.0	10^6	44.0	64.0	2.43	0.0059	0.0055	0.0092
108.0	10^4	44.0	64.0	2.31	0.53	0.49	0.83
108.0	5000	44.0	64.0	2.21	0.97	0.90	1.5
108.0	3000	43.7	64.3	2.10	1.47	1.4	2.3
108.0	2000	43.4	64.7	1.99	1.97	1.8	3.1
108.0	1000	42.6	65.5	1.75	3.05	2.8	4.7
108.0	700	41.8	66.3	1.60	3.66	3.4	5.5
108.0	500	41.0	67.1	1.45	4.21	3.9	6.3
108.0	300	39.3	68.8	1.21	4.90	4.5	7.1
108.3	200	37.6	70.7	1.04	5.38	5.0	7.6
108.3	100	36.7	71.6	0.75	5.64	5.2	7.9

Supplementary Text 10. Rectification efficiency of the diode with thick depletion layer

The rectification efficiency η at 920 MHz was evaluated with the diode using the condition to fabricate 80-nm-thick depletion layer. The observed efficiency was up to 0.07%, which is almost hundred times lower compared to the case with the diode with 35-nm-thick depletion layer. Nevertheless, this value is still almost hundred times higher compared to previous studies listed in Fig. 6e around this frequency.

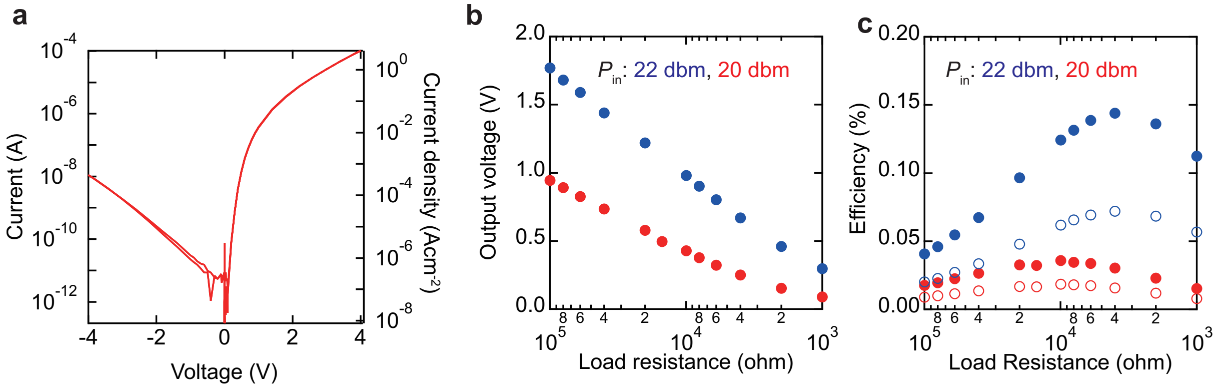

Figure S25: **Rectification efficiency of the diode with 80-nm-thick depletion layer.** (a) Current-voltage characteristics of the diode used. (b) Output voltages of the diode during rectification of a 920 MHz AC input signal. (c) Rectification efficiencies, η (open symbols) and η_{int} (filled symbols), of the diode during rectification of the 920 MHz AC input. Input power levels are indicated in the legend.

Supplementary Text 11. Comparison of static characteristics

Fig. S26 plots values of current at 2 V and capacitance for our diode using 0.5wt% P(NDIOD-T2) solution and the literatures reporting these values. The values are also listed in Table S14. Assuming that the current and capacitance are proportional to the junction area, the dashed line shows the predicted parameters when the junction area is changed in our device. This plot highlights that our diode outperforms previous reports in terms of achieving high current and small capacitance values, which are important to achieve efficient rectification at high frequencies.

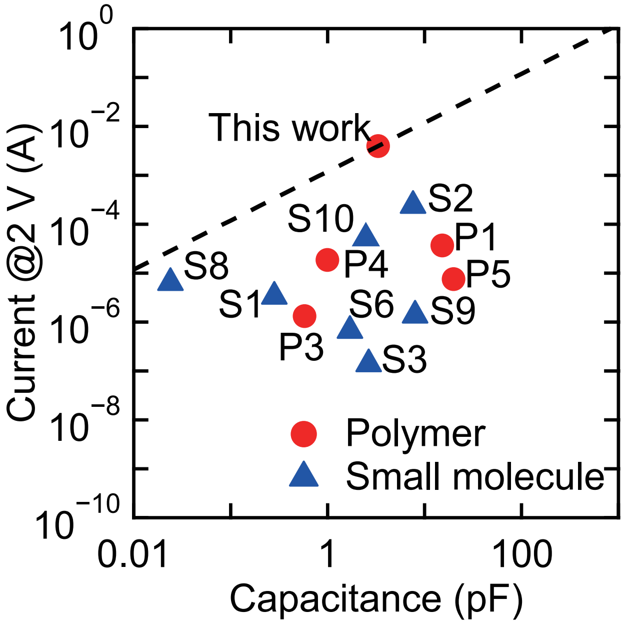

Figure S26: **Comparison of the forward current at 2 V to the literatures employing diodes based on small molecules.** (S1 (*41*), S2 (*46*), S3 (*47*), S6 (*10*), S8 (*55*), S9 (*56*)), and S10 (*57*) and polymers (P1 (*4*), P3 (*9*), P4 (*14*), and P5 (*56*)) The dashed line shows the characteristics obtained when changing the area of the diode in this study.

Table S14: **Comparison of static characteristics.** S: Area (μm^2), d: Thickness (nm), C: Capasitance (pF), I_f: Forward current at 2 V (A).

OSC	Anode	Cathode	S	d	C	I_f	Ref
Pentacene	Au	Al	1700	160	0.28	3.4×10^{-6}	S1 (*41*)
Pentacene	Au	Al	1.0×10^4	350	7.6	2.4×10^{-4}	S2 (*46*)
Pentacene	TCNQ/Cu	Al	1.0×10^4	100	2.7	1.4×10^{-7}	S3 (*47*)
Pentacene	PFBT/Au	Au	6400	100	1.7	6.7×10^{-7}	S6 (*10*)
C_6-DNTT	Pt	Ti	225	2	0.024	6.4×10^{-6}	S8 (*55*)
Pentacene	Au	Al	3.0×10^4	100	8.0	1.4×10^{-6}	S9 (*56*)
Rubrene	Au/p-doped rubrene	Al/n-doped rubrene	1.0×10^4	100	2.5	5.0×10^{-5}	S10 (*57*)
N2200	Ag	PEI/Ag	2.3×10^5	400	15	3.7×10^{-5}	P1 (*4*)
C_{16}IDT-BT	Au	Al	250	23	0.58	1.3×10^{-6}	P3 (*9*)
DPP-DTT	PEDOT:PSS	PEIE/Ag	7500	200	1.0	1.9×10^{-5}	P4 (*14*)
P3HT	Au	Al	3.0×10^4	40	20	7.5×10^{-6}	P5 (*56*)
N2200	Au	RuCp*Mes$^+$/Au	3750	20	3.3	4.0×10^{-3}	This work

REFERENCES AND NOTES

1. C. Zhao, C. G. Tang, Z.-L. Seah, Q.-M. Koh, L.-L. Chua, R.-Q. Png, P. K. Ho, Improving organic photovoltaic cells by forcing electrode work function well beyond onset of ohmic transition. *Nat. Commun.* **12**, 2250 (2021).

2. Y.-H. Kim, T.-H. Han, H. Cho, S.-Y. Min, C.-L. Lee, T.-W. Lee, Polyethylene imine as an ideal interlayer for highly efficient inverted polymer light-emitting diodes. *Adv. Funct. Mater.* **24**, 3808–3814 (2014).

3. C. G. Tang, M. C. Ang, K.-K. Choo, V. Keerthi, J.-K. Tan, M. N. Syafiqah, T. Kugler, J. H. Burroughes, R.-Q. Png, L.-L. Chua, P. K. Ho, Doped polymer semiconductors with ultrahigh and ultralow work functions for ohmic contacts. *Nature* **539**, 536–540 (2016).

4. F. A. Viola, B. Brigante, P. Colpani, G. Dell'Erba, V. Mattoli, D. Natali, M. Caironi, A 13.56 MHz rectifier based on fully inkjet printed organic diodes. *Adv. Mater.* **32**, e2002329 (2020).

5. N. Matsuhisa, S. Niu, S. J. O'Neill, J. Kang, Y. Ochiai, T. Katsumata, H.-C. Wu, M. Ashizawa, G.-J. N. Wang, D. Zhong, X. Wang, X. Gong, R. Ning, H. Gong, I. You, Y. Zheng, Z. Zhang, J. B.-H. Tok, X. Chen, Z. Bao, High-frequency and intrinsically stretchable polymer diodes. *Nature* **600**, 246–252 (2021).

6. G. Wang, F. S. Melkonyan, A. Facchetti, T. J. Marks, All-polymer solar cells: Recent progress, challenges, and prospects. *Angew. Chem. Int. Ed. Engl.* **58**, 4129–4142 (2019).

7. Z. Genene, W. Mammo, E. Wang, M. R. Andersson, Recent advances in *n*-type polymers for all-polymer solar cells. *Adv. Mater.* **31**, e1807275 (2019).

8. L. Portilla, K. Loganathan, H. Faber, A. Eid, J. G. Hester, M. M. Tentzeris, M. Fattori, E. Cantatore, C. Jiang, A. Nathan, G. Fiori, T. Ibn-Mohammed, T. D. Anthopoulos, V. Pecunia, Wirelessly powered large-area electronics for the internet of things. *Nat. Electron.* **6**, 10–17 (2023).

9. K. Loganathan, A. D. Scaccabarozzi, H. Faber, F. Ferrari, Z. Bizak, E. Yengel, D. R. Naphade, M. Gedda, Q. He, O. Solomeshch, B. Adilbekova, E. Yarali, L. Tsetseris, K. N. Salama, M. Heeney, N. Tessler, T. D. Anthopoulos, 14 GHz Schottky diodes using a *p*-doped organic polymer. *Adv. Mater.* **34**, e2108524 (2022).

10. C.-m. Kang, J. Wade, S. Yun, J. Lim, H. Cho, J. Roh, H. Lee, S. Nam, D. Bradley, J. Kim, C. Lee, 1 GHz pentacene diode rectifiers enabled by controlled film deposition on SAM-treated Au anodes. *Adv. Electron. Mater.* **2**, 1500282 (2016).

11. H. Yan, Z. Chen, Y. Zheng, C. Newman, J. R. Quinn, F. Dötz, M. Kastler, A. Facchetti, A high-mobility electron-transporting polymer for printed transistors. *Nature* **457**, 679–686 (2009).

12. J. Rivnay, M. F. Toney, Y. Zheng, I. V. Kauvar, Z. Chen, V. Wagner, A. Facchetti, A. Salleo, Unconventional face-on texture and exceptional in-plane order of a high mobility n-type polymer. *Adv. Mater.* **22**, 4359–4363 (2010).

13. S. Tatara, Y. Kuzumoto, M. Kitamura, Surface properties of substituted-benzenethiol monolayers on gold and silver: Work function, wettability, and surface tension. *Jpn. J. Appl. Phys.* **55**, 03DD02 (2016).

14. M. Yamada, Y. Takeda, S. Tokito, H. Matsui, Printed organic Schottky diode with self-assembled monolayer for 13.56 MHz band near-field communication. *Appl. Phys. Exp.* **16**, 056502 (2023).

15. Y. Lin, Y. Firdaus, F. H. Isikgor, M. I. Nugraha, E. Yengel, G. T. Harrison, R. Hallani, A. El-Labban, H. Faber, C. Ma, X. Zheng, A. Subbiah, C. T. Howells, O. M. Bakr, I. McCulloch, S. D. Wolf, L. Tsetseris, T. D. Anthopoulos, Self-assembled monolayer enables hole transport layer-free organic solar cells with 18% efficiency and improved operational stability. *ACS Energy Lett.* **5**, 2935–2944 (2020).

16. Y. Zhou, C. Fuentes-Hernandez, J. Shim, J. Meyer, A. J. Giordano, H. Li, P. Winget, T. Papadopoulos, H. Cheun, J. Kim, M. Fenoll, A. Dindar, W. Haske, E. Najafabadi, T. M. Khan, H. Sojoudi, S. Barlow, S. Graham, J.-L. Brédas, S. R. Marder, A. Kahn, B. Kippelen, A

universal method to produce low-work function electrodes for organic electronics. *Science* **336**, 327–332 (2012).

17. V. B. Engelkes, J. M. Beebe, C. D. Frisbie, Length-dependent transport in molecular junctions based on SAMs of alkanethiols and alkanedithiols: Effect of metal work function and applied bias on tunneling efficiency and contact resistance. *J. Am. Chem. Soc.* **126**, 14287–14296 (2004).

18. V. A. Kolesov, C. Fuentes-Hernandez, W.-F. Chou, N. Aizawa, F. A. Larrain, M. Wang, A. Perrotta, S. Choi, S. Graham, G. C. Bazan, T.-Q. Nguyen, S. R. Marder, B. Kippelen, Solution-based electrical doping of semiconducting polymer films over a limited depth. *Nat. Mater.* **16**, 474–480 (2017).

19. Y. Kim, S. Chung, K. Cho, D. Harkin, W.-T. Hwang, D. Yoo, J.-K. Kim, W. Lee, Y. Song, H. Ahn, Y. Hong, H. Sirringhaus, K. Kang, T. Lee, Enhanced charge injection properties of organic field-effect transistor by molecular implantation doping. *Adv. Mater.* **31**, e1806697 (2019).

20. Y. Yamashita, J. Tsurumi, M. Ohno, R. Fujimoto, S. Kumagai, T. Kurosawa, T. Okamoto, J. Takeya, S. Watanabe, Efficient molecular doping of polymeric semiconductors driven by anion exchange. *Nature* **572**, 634–638 (2019).

21. M. Ishii, Y. Yamashita, S. Watanabe, K. Ariga, J. Takeya, Doping of molecular semiconductors through proton-coupled electron transfer. *Nature* **622**, 285–291 (2023).

22. Y. Yamashita, S. Kohno, E. Longhi, S. Jhulki, S. Kumagai, S. Barlow, S. R. Marder, J. Takeya, S. Watanabe, N-type molecular doping of a semicrystalline conjugated polymer through cation exchange. *Commun. Mater.* **5**, 79 (2024).

23. M. Xiong, X.-Y. Deng, S.-Y. Tian, K.-K. Liu, Y.-H. Fang, J.-R. Wang, Y. Wang, G. Liu, J. Chen, D. R. Villalva, D. Baran, X. Gu, T. Lei, Counterion docking: A general approach to reducing energetic disorder in doped polymeric semiconductors. *Nat. Commun.* **15**, 4972 (2024).

24. X. Zhao, M. Alsufyani, J. Tian, Y. Lin, S. Y. Jeong, H. Y. Woo, Y. Yin, I. McCulloch, High efficiency n-type doping of organic semiconductors by cation exchange. *Adv. Mater.* **36**, e2412811 (2024).

25. G. M. Rangger, O. T. Hofmann, L. Romaner, G. Heimel, B. Bröker, R.-P. Blum, R. L. Johnson, N. Koch, E. Zojer, F4TCNQ on Cu, Ag, and Au as prototypical example for a strong organic acceptor on coinage metals. *Phys. Rev. B* **79**, 165306 (2009).

26. B. J. Topham, M. Kumar, Z. G. Soos, Profiles of work function shifts and collective charge transfer in submonolayer metal–organic films. *Adv. Funct. Mater.* **21**, 1931–1940 (2011).

27. A. J. Giordano, F. Pulvirenti, T. M. Khan, C. Fuentes-Hernandez, K. Moudgil, J. H. Delcamp, B. Kippelen, S. Barlow, S. R. Marder, Organometallic dimers: Application to work-function reduction of conducting oxides. *ACS Appl. Mater. Interf.* **7**, 4320–4326 (2015).

28. K. Akaike, M. V. Nardi, M. Oehzelt, J. Frisch, A. Opitz, C. Christodoulou, G. Ligorio, P. Beyer, M. Timpel, I. Pis, F. Bondino, K. Moudgil, S. Barlow, S. R. Marder, N. Koch, Effective work function reduction of practical electrodes using an organometallic dimer. *Adv. Funct. Mater.* **26**, 2493–2502 (2016).

29. A. S. Hyla, P. Winget, H. Li, C. Risko, J.-L. Brédas, Work function reduction by a redox-active organometallic sandwich complex. *Org. Electron.* **37**, 263–270 (2016).

30. I. E. Jacobs, A. J. Moulé, Controlling molecular doping in organic semiconductors. *Adv. Mater.* **29**, 1703063 (2017).

31. X. Lin, B. Wegner, K. M. Lee, M. A. Fusella, F. Zhang, K. Moudgil, B. P. Rand, S. Barlow, S. R. Marder, N. Koch, A. Kahn, Beating the thermodynamic limit with photo-activation of n-doping in organic semiconductors. *Nat. Mater.* **16**, 1209–1215 (2017).

32. B. Fan, L. Ying, P. Zhu, F. Pan, F. Liu, J. Chen, F. Huang, Y. Cao, All-polymer solar cells based on a conjugated polymer containing siloxane-functionalized side chains with efficiency over 10%. *Adv. Mater.* **29**, 1703906 (2017).

33. Y. Yamashita, S. Jhulki, D. Bhardwaj, E. Longhi, S. Kumagai, S. Watanabe, S. Barlow, S. R. Marder, J. Takeya, Highly air-stable, *n*-doped conjugated polymers achieved by dimeric organometallic dopants. *J. Mater. Chem. C* **9**, 4105–4111 (2021).

34. J.-K. Tan, R.-Q. Png, C. Zhao, P. K. Ho, Ohmic transition at contacts key to maximizing fill factor and performance of organic solar cells. *Nat. Commun.* **9**, 3269 (2018).

35. B. De Boer, A. Hadipour, M. M. Mandoc, T. Van Woudenbergh, P. W. Blom, Tuning of metal work functions with self-assembled monolayers. *Adv. Mater.* **17**, 621–625 (2005).

36. R. Schlesinger, F. Bianchi, S. Blumstengel, C. Christodoulou, R. Ovsyannikov, B. Kobin, K. Moudgil, S. Barlow, S. Hecht, S. R. Marder, F. Henneberger, N. Koch, Efficient light emission from inorganic and organic semiconductor hybrid structures by energy-level tuning. *Nat. Commun.* **6**, 6754 (2015).

37. A. Jablonski, J. Zemek, Overlayer thickness determination by XPS using the multiline approach. *Surf. Interf. Anal.* **41**, 193–204 (2009).

38. R. C. Shallcross, T. Stubhan, E. L. Ratcliff, A. Kahn, C. J. Brabec, N. R. Armstrong, Quantifying the extent of contact doping at the interface between high work function electrical contacts and poly(3-hexylthiophene) (P3HT). *J. Phys. Chem. Lett.* **6**, 1303–1309 (2015).

39. I. Lange, J. C. Blakesley, J. Frisch, A. Vollmer, N. Koch, D. Neher, Band bending in conjugated polymer layers. *Phys. Rev. Lett.* **106**, 216402 (2011).

40. M. Raja, B. Eccleston, The significance of Debye length in disordered doped organic devices. *J. Appl. Phys.* **110**, 114524 (2011).

41. S. Steudel, K. Myny, V. Arkhipov, C. Deibel, S. De Vusser, J. Genoe, P. Heremans, 50 MHz rectifier based on an organic diode. *Nat. Mater.* **4**, 597–600 (2005).

42. A. Chasin, M. Nag, A. Bhoolokam, K. Myny, S. Steudel, S. Schols, J. Genoe, G. Gielen, P. Heremans, Gigahertz operation of a-IGZO Schottky diodes. *IEEE Trans. Electron. Dev.* **60**, 3407–3412 (2013).

43. K. Fujimori, K. Kobayashi, “Optimal design method of RF-DC conversion circuits for various input and load conditions required for rectenna site design,” in *2022 16th European Conference on Antennas and Propagation (EuCAP)* (IEEE, 2022), pp. 1–4.

44. M. Nikolka, K. Broch, J. Armitage, D. Hanifi, P. J. Nowack, D. Venkateshvaran, A. Sadhanala, J. Saska, M. Mascal, S.-H. Jung, J.-K. Lee, I. McCulloch, A. Salleo, H. Sirringhaus, High-mobility, trap-free charge transport in conjugated polymer diodes. *Nat. Commun.* **10**, 2122 (2019).

45. H.-I. Un, S. A. Gregory, S. K. Mohapatra, M. Xiong, E. Longhi, Y. Lu, S. Rigin, S. Jhulki, C.-Y. Yang, T. V. Timofeeva, J.-Y. Wang, S. K. Yee, S. Barlow, S. R. Marder, J. Pei, Understanding the effects of molecular dopant on *n*-type organic thermoelectric properties. *Adv. Energy Mater.* **9**, 1900817 (2019).

46. K. Myny, S. Steudel, P. Vicca, J. Genoe, P. Heremans, An integrated double half-wave organic Schottky diode rectifier on foil operating at 13.56 MHz. *Appl. Phys. Lett.* **93**, (2008).

47. H. Wang, Z. Ji, L. Shang, X. Liu, Y. Peng, M. Liu, Interface effect on the performance of rectifier based on organic diode. *IEEE Electron. Dev. Lett.* **31**, 506–508 (2010).

48. D. Im, H. Moon, M. Shin, J. Kim, S. Yoo, Towards Gigahertz operation: Ultrafast low turn-on organic diodes and rectifiers based on C_{60} and tungsten oxide. *Adv. Mater.* **23**, 644–648 (2011).

49. H. Kleemann, S. Schumann, U. Jörges, F. Ellinger, K. Leo, B. Lüssem, Organic pin-diodes approaching ultra-high-frequencies. *Org. Electron.* **13**, 1114–1120 (2012).

50. J. Semple, S. Rossbauer, C. H. Burgess, K. Zhao, L. K. Jagadamma, A. Amassian, M. A. McLachlan, T. D. Anthopoulos, Radio frequency coplanar ZnO Schottky nanodiodes processed from solution on plastic substrates. *Small* **12**, 1993–2000 (2016).

51. S. Evans, Correction for the effects of adventitious carbon overlayers in quantitative XPS analysis. *Surf. Interf. Anal.* **25**, 924–930 (1997).

52. H. Shinotsuka, S. Tanuma, C. J. Powell, D. R. Penn, Calculations of electron inelastic mean free paths. X. Data for 41 elemental solids over the 50 eV to 200 keV range with the relativistic full Penn algorithm. *Surf. Interf. Anal.* **47**, 871–888 (2015).

53. S. Tanuma, C. J. Powell, D. R. Penn, Calculations of electron inelastic mean free paths. V. Data for 14 organic compounds over the 50–2000 eV range. *Surf. Interf. Anal.* **21**, 165–176 (1994).

54. X. Yang, J. Liu, L. J. A. Koster, The exceptionally high dielectric constant of doped organic semiconductors. *Adv. Electron Mater.* **11**, 2400413 (2025).

55. Y. Li, J. Xie, L. Sun, J. Zeng, L. Zhou, Z. Hao, L. Pan, J. Ye, P. Wang, Y. Li, J.-B. Xu, Y. Shi, X. Wang, D. He, Monolayer organic crystals for ultrahigh performance molecular diodes. *Adv. Sci.* **11**, e2305100 (2024).

56. K. Ferchichi, S. Pecqueur, D. Guerin, R. Bourguiga, K. Lmimouni, Organic doped diode rectifier based on parylene-electronic beam lithogrpahy process for radio frequency applications. *Org. Electron.* **97**, 106266 (2021).

57. M. F. Sawatzki, H. Kleemann, B. K. Boroujeni, S.-J. Wang, J. Vahland, F. Ellinger, K. Leo, Doped highly crystalline organic films: Toward high-performance organic electronics. *Adv. Sci.* **8**, 2003519 (2021).
